# Regulation of melanin production in fungi

**DOI:** 10.3389/ffunb.2025.1621764

**Published:** 2025-08-29

**Authors:** Kamaldeep Chhoker, Georg Hausner, Steven D. Harris

**Affiliations:** ^1^ Department of Biological Sciences, University of Manitoba, Winnipeg, MB, Canada; ^2^ Department of Microbiology, University of Manitoba, Winnipeg, MB, Canada; ^3^ Department of Plant Pathology, Entomology and Microbiology, Iowa State University, Ames, IA, United States

**Keywords:** fungal melanin, benefits of melanin, melanin biosynthesis, melanin biosynthetic pathways, transcription factors (TFs)

## Abstract

Melanin is a dark macromolecule found in organisms ranging from animals to fungi and plants. In fungi, melanin is a secondary metabolite that is not essential per se for growth but does provide various benefits that facilitate adaptation to stressful conditions such as UV light, desiccation, oxygen radicals, and extreme temperatures. The biosynthetic pathways of most types of melanin are known and documented, but the regulation of those pathways is not well understood. In fungi, known pathways for melanin production include those directing the synthesis of 1,8-DHN melanin and L-DOPA melanin, as well as the tyrosine degradation pathway. Genetic studies have identified structural genes and enzymes that play a role in these different melanin biosynthesis pathways. Recent studies have focused on the roles of various transcription factors (TFs) and signaling circuits (e.g., cAMP/PKA and the HOG pathway) in regulating the expression of the biosynthetic pathways. The review will provide insights into what is known about these TFs and regulatory circuits in diverse fungi in an attempt to identify common themes.

## 1 Introduction

Melanin is a dark, multifunctional pigment that is produced via the oxidative polymerization of phenolic and indolic compounds (Gomez and Nosanchuk, 2003; Solano, 2014; Suwannarach et al., 2019). The word melanin is derived from the Greek word “melanos,” meaning “black” or “very dark” (Riley, 1997; Gessler et al., 2014). The term melanin was first used by the Swedish chemist Berzelius to name a dark pigment extracted from eye membranes in 1840 (Borovanský, 2011). Melanin does not refer to a single substance but a group of substances that have similar properties (Bell and Wheeler, 1986; Butler and Day, 1998; Langfelder et al., 2003). Because they originate from different starting precursors, melanin particles can be found in a range of shapes and sizes that include rods, platelets, and planar arrays (Glass et al., 2012; Song et al., 2023). Based on the chemical precursor and the biosynthetic pathway, melanin pigments are classified into five different types: eumelanin, pheomelanin, neuromelanin, allomelanin, and pyomelanin (Ambrico, 2016; Cao et al., 2021). These starting precursors condense and polymerize into nanometer- to micron-size particles (Hong et al., 2012, 2018). Eumelanin, pheomelanin, and neuromelanin are found associated with animal tissues, whereas allomelanin and pyomelanin are found mostly in bacteria, fungi, and plants (Xie et al., 2019). Melanins are insoluble hydrophobic pigments that are negatively charged and have high molecular weight (Nosanchuk and Casadevall, 2003; Paolo et al., 2006; Solano, 2014). Another key feature of melanin is the presence of a stable free radical population (Sealy et al., 1982).

The coloration of melanin can vary, ranging from mainly dark brown to black, but in some instances, red or yellow coloration is also observed (Solano, 2014). Features that distinguish melanin from other secondary compounds such as carotenoids and polyketides include the following: i) melanin is extremely heat resistant and can withstand temperatures up to 600°C (Gallas et al., 2000), and ii) it is highly insoluble and resistant to strong acids, detergents, and reducing agents but is soluble in bases and phenols (Jacobson, 2000). Because of these features, the structure is quite hard to identify since classical methods using aqueous or organic fluids end up disrupting its organization (Nosanchuk et al., 2015). Like all naturally occurring pigments such as carotenoids, chlorophyll, and flavonoids, melanin contains conjugate moieties, such as aromatic rings, that allow electronic resonance and mediate energy transfer reactions (Cordero and Casadevall, 2017). Benefits and applied uses of melanin have been reviewed thoroughly (Cordero and Casadevall, 2017; Tran-Ly et al., 2020; Mattoon et al., 2021; Suthar et al., 2023). Although the biosynthetic pathways that produce melanin are reasonably well understood (Eisenman and Casadevall, 2011; Suthar et al., 2023; Qin and Xia, 2024), much less is known about the regulation that ensures the proper timing and location of production. Recent studies have implicated the PKA and HOG pathways as key signaling components of this regulation, while also identifying transcription factors that control the expression of the biosynthetic pathways. This review focuses on these recent advances and highlights remaining issues.

## 2 Fungal melanin

In general, fungal melanin is typically found in the outer regions of the cell wall though it can also be found clustered on the cell wall surface (Bayry et al., 2014). In some fungi, melanin acts as a structural component of spores, providing protection against various environmental stresses, increasing the survivability of the fungi (Xu et al., 2022). Melanin in many fungi is formed by a complex of differentially sized spherical particles that are approximately 200 nm in diameter and are known as melanin granules (Franzen et al., 2006; Kogej et al., 2007). These granules are composed of fungal melanosomes, which range from 30 to 120 nm in diameter. Melanin granules allow macromolecules to pass through the melanin, meaning that there are pores present in the melanin layers. Depending on the species, melanin tends to be stacked in layers with pores ranging between 1 and 4 nm in diameter to facilitate the passage of macromolecules (Eisenman et al., 2005; Casadevall et al., 2012). Since the cell structures of different fungi vary in their organization and materials, so does the stacking distance of the melanin layers; examples include 4.15 Å for *Exophiala dermatitidis* (also known as *Wangiella dermatitidis*), 4.45 Å for *Aspergillus niger*, and 4.39 Å for *Cryptococcus neoformans* (Nosanchuk et al., 2015). The indolic and/or phenolic monomers are ordered into planar arrangements of regularly interspaced stacked layers similar to graphite (Kim et al., 2016), and these layers can then cross-link into a more heterogeneous macromolecular configuration. This pattern of stacking is known as local-order-global-disorder and involves a combination of π-stacking, hydrogen, and ionic-bonded nanostructures with the melanin granules (Meredith and Sarna, 2006; Nosanchuk et al., 2015; Kim et al., 2016).

As the synthesis of melanin produces various highly reactive and toxic intermediates, fungal melanization occurs in specialized sphingolipid-enriched vesicles termed melanosomes, which are generally similar to mammalian melanosomes (Walker et al., 2010; Upadhyay et al., 2016). These vesicles contain laccase enzymes, leading to supramolecular buildup of melanin particles that are retained within the cell wall (Seiji et al., 1963; Camacho et al., 2019). The vesicles mediate the transport of intercellularly synthesized macromolecules to targeted sites on the cell surface where they can be captured by the cell wall (Eisenman et al., 2009). Studies have revealed that the melanin polymer is covalently bonded to cell wall chitin and is also found associated with other cellular moieties, including polysaccharides such as chitosan and plasma membrane-derived lipids (Zhong et al., 2008; Chatterjee et al., 2015). Evidence of this has also been provided whereby mutations affecting chitin synthesis genes in different fungal species, such as *E. dermatitidis*, *C. neoformans*, and *Candida albicans*, lead to a “leaky melanin” phenotype such that strains are able to synthesize melanin but the cell wall is unable to retain the melanin granules, which end up leaking into the extracellular space (Wang et al., 1999; Banks et al., 2005; Walton et al., 2005; Baker et al., 2007; Tsirilakis et al., 2012). Conversely, an increase in cell wall chitin or chitosan content reportedly increases melanin deposition (Banks et al., 2005; Baker et al., 2007; Tsirilakis et al., 2012). During the budding process of melanized yeasts, melanosomes in the cell wall are degraded or displaced, allowing daughter cells to emerge (Nosanchuk and Casadevall, 2003; Eisenman et al., 2005). Much of the work in understanding melanin structure and morphology has been performed on the so-called melanin “ghosts,” which are macromolecular structures obtained after hot acid digestion of melanized cells (Eisenman et al., 2009; Chatterjee et al., 2015). Melanin ghosts are composed of smaller melanin granules that are arranged in concentric layers embedded within the fungal cell wall (Eisenman et al., 2005). Melanin produced by fungi varies depending on the species that produces it. Most ascomycetes produce 1,8-DHN melanin via the polyketide synthase pathway (Nosanchuk et al., 2015); another type of melanin, called L-DOPA melanin, is produced mainly by basidiomycetes (Nosanchuk et al., 2015). Species such as *Aspergillus fumigatus* and *A. niger* can produce multiple different melanin types, which presumably can act as a failsafe during stressful conditions if certain nutrient requirements are not met (Pukkila-Worley et al., 2005). Some species such as *E. dermatitidis* have homologs of genes involved in 1,8-DHN melanin, L-DOPA melanin, and L-tyrosine degradation melanin pathways, but the exact mechanism or conditions that can trigger the production of L-DOPA or L-tyrosine melanin are not known (Paolo et al., 2006; Chen et al., 2014).

## 3 Fungal melanin biosynthesis

The main type of melanin produced by fungi, especially by ascomycetes, is 1,8-dihydroxynaphthalene (DHN) melanin via the polyketide synthase pathway (Nosanchuk and Casadevall, 2015). 1,8-DHN melanin is named after one of the pathway intermediates, 1,8-dihydroxynaphthalene, which was first identified in 1976 (Stipanovic and Bell, 1976). The second type of melanin, L-DOPA melanin, is named after one of the precursors, L-3,4-dihydroxyphenylalanine (Hamilton and Gomez, 2002). Besides the polymerization of 1,8-DHN, different species can also utilize other pigment precursors such as tyrosine, gamma-glutaminyl-4-hydroxybenzene (GHB), catechol, homogentisic acid, and scytalone (Bell et al., 1976; Weijin et al., 2013; Belozerskaya et al., 2016). The synthesis of eumelanin is catalyzed by phenoloxidases from L-DOPA substrates by fungal species such as *C. neoformans* (Langfelder et al., 2003; Nosanchuk and Casadevall, 2015; Tran-Ly et al., 2020). L-DOPA melanin is mainly synthesized by basidiomycetes, which occasionally also produce glutaminyl-3,4-dihydroxybenzene (GDHB) melanin (Henson et al., 1999; Selvakumar et al., 2008).

There are many fungal species that do not produce melanin under normal circumstances, but when supplemented with DOPA, they tend to produce L-DOPA melanin (Butler et al., 1989; Butler and Day, 1998). Despite the nature of the precursor, all fungal melanins tend to share similarities in functional groups and physiochemical properties (Fogarty and Tobin, 1996). During synthesis, several enzymes, such as tyrosinase, laccase, and catechol oxidase, carry out the rate-limiting initial oxidation of the starting phenolic precursors (Eisenman and Casadevall, 2012; D’Ischia et al., 2013; Solano, 2014), and the activity of these enzymes depends on the copper ions present at the catalytic site (Mauch et al., 2013; Upadhyay et al., 2013).

In fungi, the three different categories of melanin include 1,8-DHN melanin (allomelanin and pyomelanin), L-DOPA melanin (eumelanin and pheomelanin), and GHB melanin.

### 3.1 1,8-DHN melanin (allomelanin and pyomelanin)

**Figure 1:**
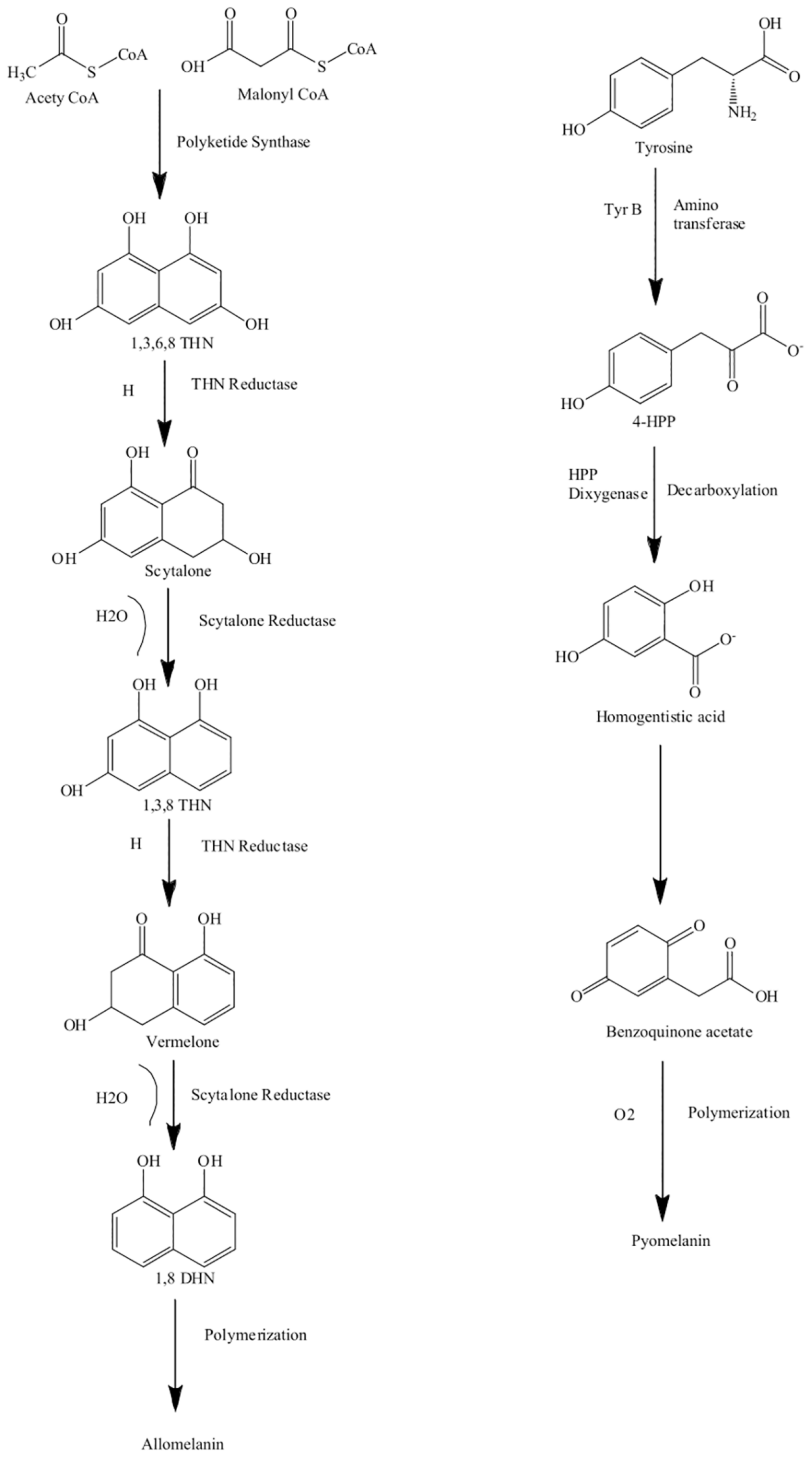
Biosynthesis pathway of allomelanin (1,8-DHN melanin) via the polyketide synthase (PKS) and pyomelanin derived from homogentisic acid. Pathways adapted from Suthar et al. (2023).

The word “allo” refers to the Greek prefix meaning “heterogeneous” or “different” (Cao et al., 2021). The precursors of allomelanin can vary such that, depending on the precursor, allomelanins are referred to as 1,8-DHN melanin, HPQ melanin, or catechol melanin (Funa et al., 2005). The starting precursor for the synthesis of 1,8-DHN melanin, malonyl-CoA, was first identified by Fujii et al. (2000) in *Colletotrichum lagenarium*. Another precursor of 1,8-DHN melanin is acetyl-CoA, and both malonyl-CoA and acetyl-CoA are produced endogenously (Nosanchuk et al., 2015). These starting precursors are converted by polyketide synthase (PKS) to the first detectable intermediate 1,3,6,8-tetrahydroxynaphthalene (1,3,6,8-THN). 1,3,6,8-THN is reduced by hydroxynaphthalene reductase to produce scytalone (Alspaugh et al., 1997; Thompson et al., 2000). Scytalone is dehydrated enzymatically to 1,3,8-trihydroxynaphthalene (Alspaugh et al., 1998), which is then further reduced by a second reductase to vermelone (Basarab et al., 1999; Thompson et al., 2000). Vermelone is then further dehydrated by scytalone dehydratase to form the next intermediate 1,8-dihydroxynaphthalene (1,8-DHN). The pathway then involves a series of steps, including a dimerization of the 1,8-DHN molecules, followed by polymerization catalyzed by a laccase (Bloomfield and Alexander, 1967). 1,8-DHN proceeds through a C-C coupling reaction of the naphthalene rings, giving three 1,8-DHN dimers, which are then further oxidized to form a mixture of longer oligomers, which self-assemble to form the melanin structure (**Figure 1**) (Cecchini et al., 2017; Manini et al., 2018). The structure of the 1,8-DHN melanin polymer is not well known, but a study conducted by Beltran-Garcia et al. (2014) observed the presence of 50 1,8-DHN units in the polymer of melanin in the mycelium of *Mycosphaerella fijiensis*. The polyketide synthase responsible for the production of 1,8-DHN melanin generally possesses a similar structure across fungi, including a β-ketosynthase domain (β-KS), an acyl transferase domain (AT), and an acyl carrier domain (ACP). These are sometimes followed by a thiosterase domain (TE), which is responsible for detaching the polyketides from the enzyme (Watanabe et al., 2000; Fujii et al., 2001). 1,8-DHN melanin production can be inhibited by tricyclazole, pyroquilone, phthalide, and clobenthiazone (Selvakumar et al., 2008).

Like allomelanins, pyomelanins are derived from the oxidative polymerization of nitrogen-free precursors such as homogentisic acid (HGA) (Funa et al., 2005; Seo and Choi, 2020). Pyomelanin originates from the catabolism of either tyrosine or phenylalanine. The enzyme 4-hydroxyphenylpyruvic acid dioxygenase (HPPD) catalyzes the conversion of 4-hydroxyphenylpyruvate to HGA. Pyomelanin is generated through autooxidation to form benzoquinone acetic acid, which is then self-polymerized to form HGA and the pyomelanin polymer (Turick et al., 2010; Keller et al., 2011). Pyomelanin polymers tend to be smaller compared to other melanin pigments (**Figure 1**).

### 3.2 L-DOPA melanin (eumelanin and pheomelanin)

**Figure 2:**
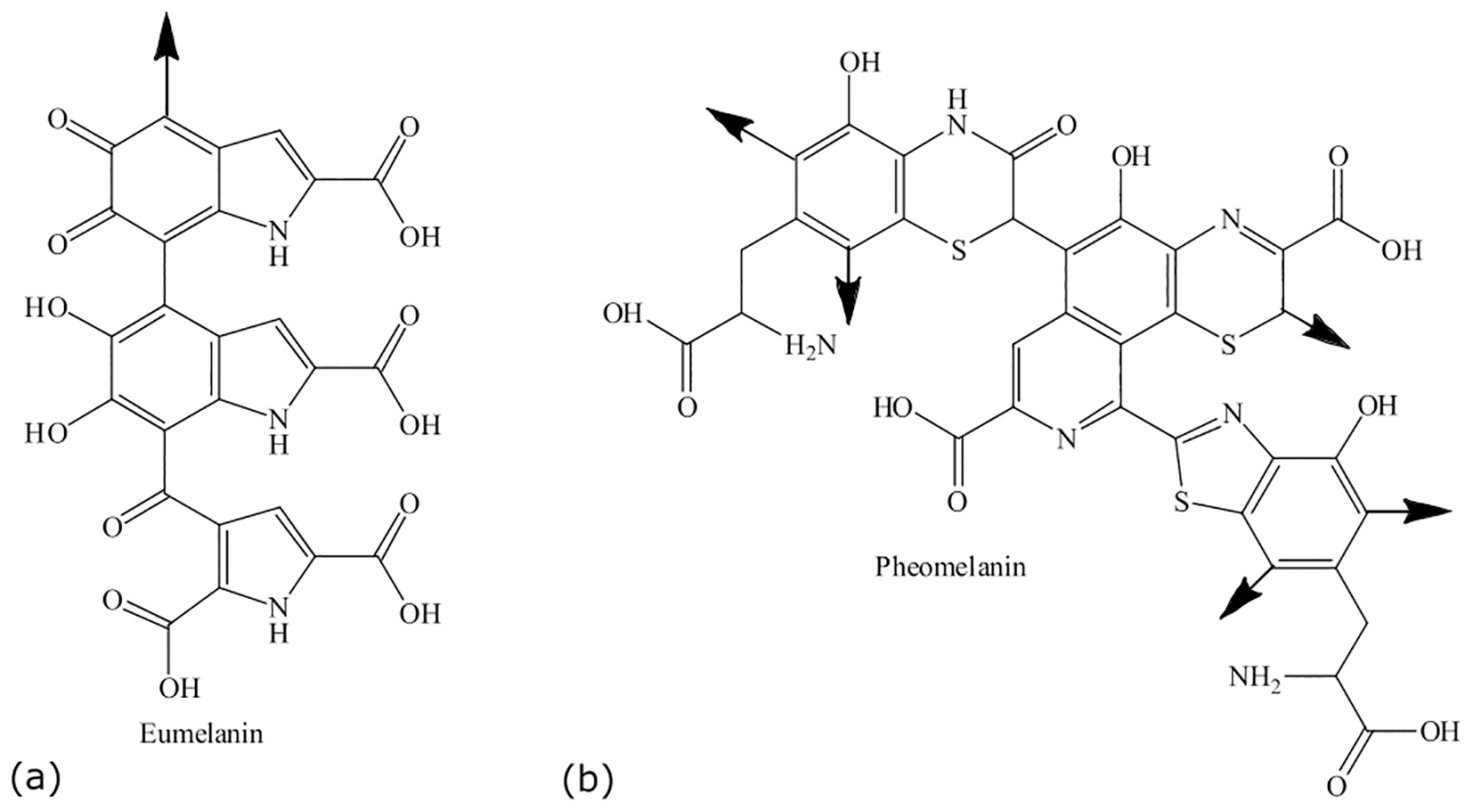
Structures of eumelanin **(a)** and pheomelanin **(b)**. Adapted from Sansinenea and Ortiz (2015).

**Figure 3:**
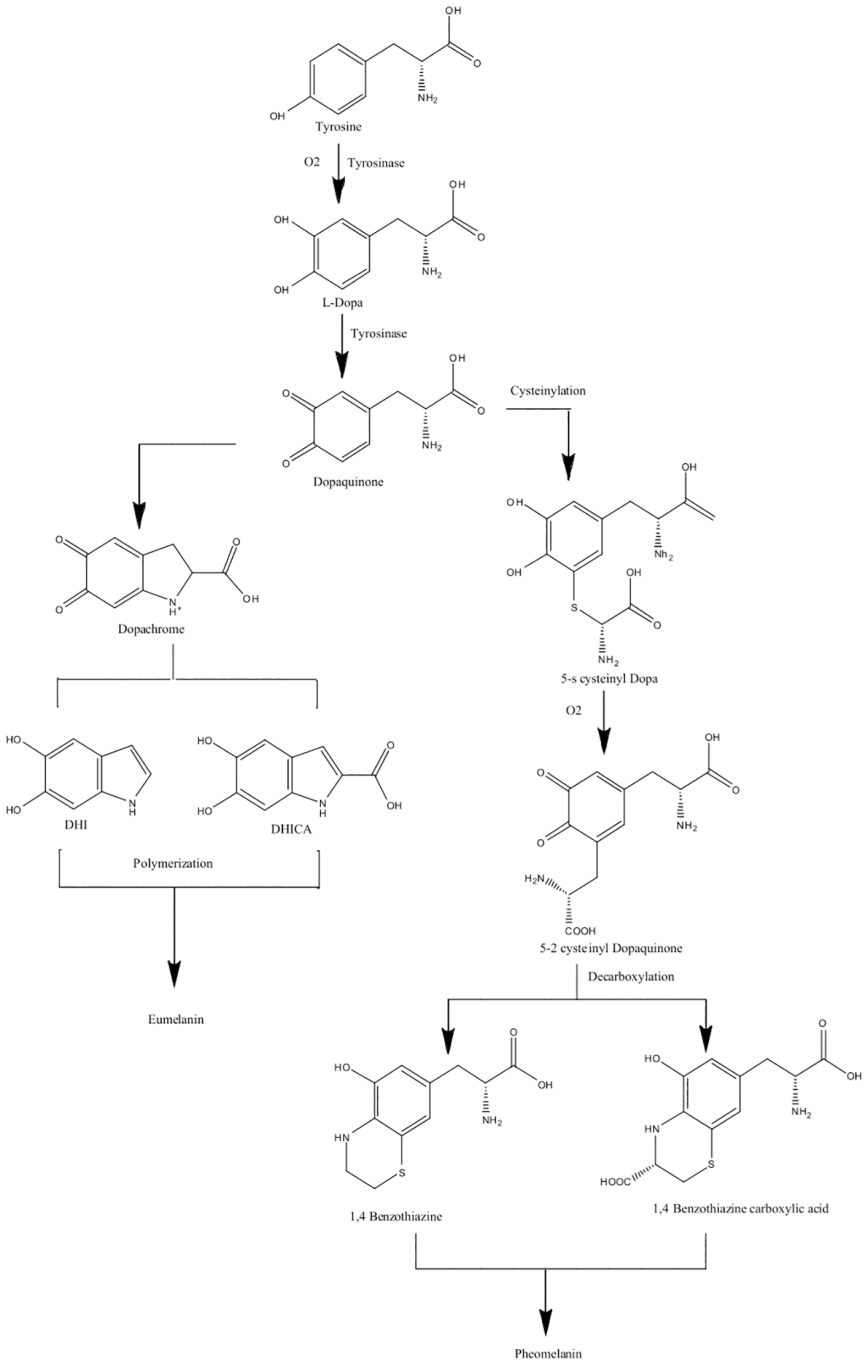
Biosynthesis pathway of eumelanin and pheomelanin (L-DOPA melanins) using tyrosinase enzymes. Pathways adapted from Suthar et al. (2023).

“Eu” is the Greek word for “good” or “well,” and pheo means “dark” in ancient Greek (Cao et al., 2021). The main difference between eumelanin and pheomelanin is attributed to the potential of eumelanin (**Figure 2a**) to act as a photoprotector and the phototoxic nature of pheomelanin (**Figure 2b**) (Cao et al., 2021). Pheomelanins are also believed to contain benzothiazine subunits that are synthesized from L-DOPA and cysteine (Simon and Peles, 2010). Both eumelanin and pheomelanin are comprised of repeating units linked by carbon–carbon bonds (Costin and Hearing, 2007). Phenoloxidases for L-DOPA melanin can either be laccases or tyrosinases, both of which have copper ligands and require copper ions for activity. Both play different roles: laccases catalyze the one-step oxidation of dihydroxy phenols to quinones, and tyrosinases catalyze the two-step oxidation of tyrosine (Langfelder et al., 2003). In brief, the biosynthesis of eumelanin (**Figure 3**) begins with tyrosine, which is oxidized by oxygen, followed by tyrosinase that forms levodopa (L-DOPA) and then dopaquinone (Simon and Peles, 2010; Cao et al., 2021). During the L-DOPA melanin pathway, hydroxylation of L-tyrosine to dopaquinone or the oxidation of L-DOPA to dopaquinone is catalyzed by tyrosinase or laccase, respectively (Pomerantz and Warner, 1967). If there are no thiol groups present, dopaquinone forms leucodopachrome, which is then oxidized to dopachrome. Hydroxylation and decarboxylation then yield dihydroxyindoles, which are then further polymerized to form L-DOPA melanin (Ozeki et al., 1997a, 1997b; Butler and Day, 1998; Williamson, 1994). The synthesis of L-DOPA melanin has been shown to be inhibited by tropolone, kojic acid, and diethyldithiocarbamate (Salgado-Castillo et al., 2023).

### 3.3 GHB melanin

**Figure 4:**
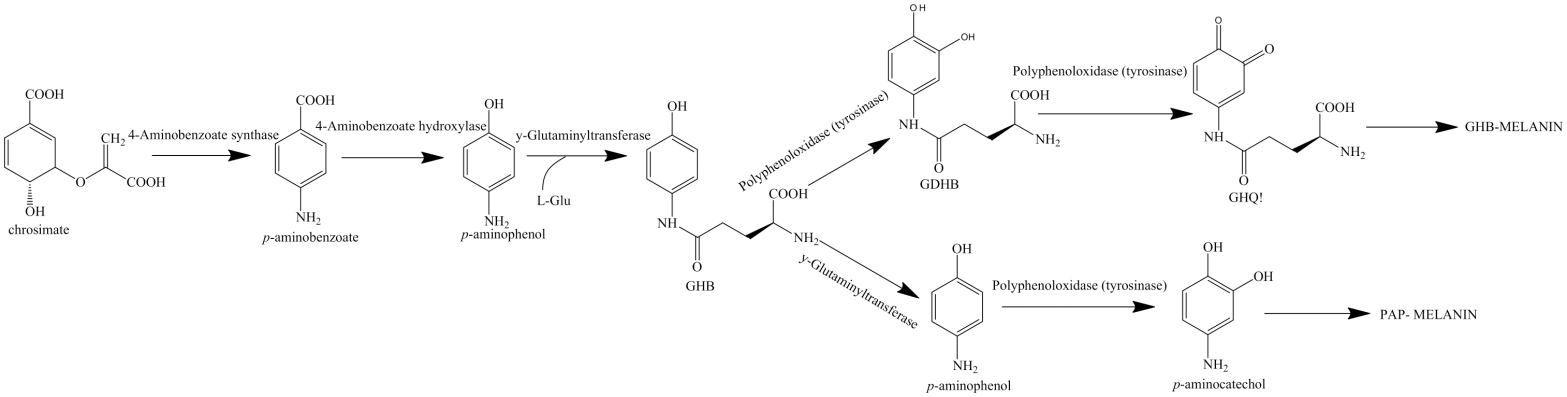
Biosynthesis pathway for GHB melanin using chorismate as a precursor. Pathway adapted from Weijin et al. (2013).

In mushrooms, such as *Agaricus bisporus*, another type of melanin referred to as GHB is present. γ-L-glutaminyl 4-hydroxybenzene (GHB) is found in the mycelium and the fruiting body of *A*. *bisporus*, whereas γ-L-glutaminyl-3,4_dydroxybenzene (GDHB) is found specifically in the reproductive hyphae (Stüssi and Rast, 1981). GHB melanin is also sometimes referred to as PAP melanin, where the initial substrate is *p-*aminophenol and the glutamyl groups are later removed before polymerization (Solano, 2014). GHB melanin is formed from either phenolic precursors or GHB via the action of a tyrosinase (**Figure 4**) (Weijin et al., 2013). Chorismate, which acts as the initial aromatic ring, is converted to *p-*aminophenol and conjugated with a glutamyl residue to form GHB. GHB can then be further oxidized to form glutaminyl-3,4-dihydroxybenzene (GDHB) or o-quinone (GBQ). The glutamyl residues are removed from the final pigment (Bisko et al., 2007).

## 4 Genes involved in melanin synthesis pathways

The identification of the genes involved in the production of 1,8-DHN melanin and L-DOPA melanin has been achieved using gene knockout strategies (Schumacher, 2016; Zhang et al., 2019; Nambu et al., 2021; Yang et al., 2022). The 1,8-DHN melanin synthesis gene cluster is conserved in many fungi (Schumacher, 2016; Ebert et al., 2018). The DHN melanin pathway in *A. fumigatus* is comprised of a cluster of six genes, namely, *abr1*, *abr2*, *ayg1*, *arp1*, *arp2*, and *pksP* (Perez-Cuesta et al., 2020). Pyomelanin synthesis is related to the L-tyrosine degradation pathway that includes a cluster of six genes: *hppD*, *hmgX*, *hmgA*, *fahA*, *maiA*, and *hmgR*. In *A. fumigatus*, the 1,8-DHN melanin biosynthetic gene cluster spans roughly 10 kb (Tsai et al., 1999). Genes encoded within this cluster are responsible for different steps of the 1,8-DHN melanin biosynthetic pathway. The PKS gene *alb1*, also referred to as *pksP*, participates in the β-keotacyl condensation of malonyl-CoA and acetyl-CoA to generate 2,5,6,8-tetrahydroxy-2-methyl-2,3-dihydro-4H-naphtho(2,3-b)pyran-4-one (YWA1) (Gao et al., 2022). During the second step, the *ayg1* gene hydrolyzes YWA1 to generate 1,3,6,8-tetrahydroxynaphthalene (1,3,6,8-THN). There are multiple reduction steps followed by aromatization/dehydration reactions that lead to oxidative polymerization (Perez-Cuesta et al., 2020). 1,3,6,8-THN is reduced to scytalone by the hydroxynaphthalene reductase gene *arp2* and the enzyme 1,3,6,8-reductase. The scytalone reductase gene *arp1* is responsible for the dehydration of scytalone to 1,3,8-trihydroxynaphthalene, which is followed by another reduction step by the hydroxynaphthalene reductase gene *arp2* that reduces 1,3,8-trihydroxynaphthalene to vermelone. Vermelone is then dehydrated by a multicopper oxidase gene *abr1*, which converts it to 1,8-dihydroxynaphthalene (1,8-DHN), which is polymerized into 1,8-DHN melanin by a laccase encoded by the putative laccase *abr2* gene (Perez-Cuesta et al., 2020). Various studies have looked at the functions of the genes involved in the 1,8-DHN pathway, and the results for some of them are summarized in **Table 1**.

**Table 1:** Genes involved in fungal melanin biosynthesis.

Gene	Protein	Organism	Function	Reference
*PKS/alb1* *PKS 12* and *PKS 13* *WdPKS1*	Polyketide Synthase	*A. fumigatus* *B. cinerea* *E. dermatitidis*	Production of 1,3,6,8-tetrahydroxynaphthalene (T4HN)	Gao et al., 2022 Schumacher, 2016 Paolo et al., 2006
*Ayg1* *BRN1* and *BRN2*	Abhydrolase	*A. fumigatus* *M. laxa*, *M. fructicola*, *M.fructigena*	Reduction of 1,3,6,8 THN to scytalone	Gao et al., 2022 Verde-Yanez et al., 2023
*arp1* *AISCD1* and *AISCD2* *SCD1*	Scytalone dehydratase	*A. fumigatus* *Ascochyta lentis* *M. laxa, M. laxa, M. fructicola*, *M. fructigena*	*Reduction of scytalone to 1,3,8-THN*	Gao et al., 2022 Debler and Henares, 2020 Verde-Yanez et al., 2023
*arp2*	Hydroxynaphthalene reductase	*A. fumigatus*	*Reduction of 1,3,8-THN to vermelone*	Gao et al., 2022
*abr1*	Vermelone dehydratase	*A. fumigatus*	Vermelone converted to 1,8-DHN	Gao et al., 2022 Upadhyay et al., 2013
*abr2* *pbrB*	Oxydase	*A. fumigatus* *Talaromyces marneffei*	Polymerization of 1,8-DHN to 1,8-DHN melanin	Gao et al., 2022 Upadhyay et al., 2013 Sapmak et al., 2015
*Tat*	Tyrosine aminotransferase	*A. fumigatus*	Converts tyrosine to 4-hydroxyphenylpyruvate	Schmaler-Ripcke et al., 2009 Keller et al., 2011
*hppD*	4-hydroxyphenylpyruvate dioxygenase	*A. fumigatus* *A. niger*	Catalyzes precursor of pyomelanin, HGA	Schmaler-Ripcke et al., 2009 Koch et al., 2023
*hmgA*	Homogentisate dioxygenase	*A. fumigatus* *A. niger*	Degrades HGA to 4-Maleyl acetoacetate	Schmaler-Ripcke et al., 2009 Koch et al., 2023
*fahA*	Fumarylacetoacetate hydrolase	*A. fumigatus*	Degrades HGA to Fumarate	Heinekamp et al., 2013
*maiA*	Maleylacetoacetate isomerase	*A. fumigatus*	Degrades HGA to Acetoacetate	Heinekamp et al., 2013
*melC2*	Tyrosinase	*Streptomyces lincolnensis*	oxidize L-DOPA to generate melanin	Endo et al., 2001

## 5 Benefits of melanin

Melanin provides various benefits to organisms that produce it. One key aspect that distinguishes melanin from other natural chromophores is its ability to absorb every wavelength of light (Riesz et al., 2006; Cordero et al., 2018). Melanin is not essential for the growth of fungi, but it does facilitate the ability to survive harmful conditions. The various benefits of melanin are reviewed in detail elsewhere (Cordero and Casadevall, 2017; Suthar et al., 2023) and summarized in **Table 2**.

**Table 2:** Benefits of melanin in fungi.

Benefits of Melanin in fungi	Reference
Protection from UV stress	Natarajan et al., 2014
Protection from various environmental stresses in fungi	Salgado-Castillo et al., 2023
Protection against microbes in fungi	Nosanchuk and Casadevall, 2003
Protection against oxidative stress	Rosas and Casadevall, 2006; Gorbushina et al., 2008; Kejžar et al., 2013; Cordero et al., 2018; Meredith and Sarna, 2006
Protection against enzymatic lysis	Rosas and Casadevall, 2001; Lin and Chen, 2005
Protection against radiation	Gessler et al., 2014; Pacelli et al., 2017; El-Bialy et al., 2019;
Provides structural support to the appressorium in plant pathogenic fungi	Belozerskaya et al., 2016; Chethana et al., 2021; Li et al., 2022

## 6 Applied uses of fungal melanin

For industrial use, eumelanins are preferred over allomelanins, since allomelanins are attached to the inner side of the fungal cell wall, making the extraction process more challenging (Mattoon et al., 2021). Over the years, extraction protocols have been modified, resulting in significant improvements in melanin yields. Examples include *Auricularia auricula*, where 10% of the biomass consisted of melanin following treatment with lytic enzymes, guanidinium thiocyanate, chloroform, and HCl (Prados-Rosales et al., 2015), and *Armillaria cepistipes*, in which a 99% yield increase (27.98 g/L) was obtained using simpler extraction procedures (Ribera et al., 2019). Besides extraction protocols, nutrient composition, temperature, and pH have also been shown to play a part in melanin yield (Qin and Xia, 2024). Recent studies have also examined the overexpression of tyrosinase genes and their impact on melanin production (Tran-Ly et al., 2020; Mattoon et al., 2021). Other recent studies have also identified new *Exophiala* species that excrete melanin, thereby simplifying the extraction process (Carr et al., 2023). Ultimately, insights into the regulatory pathways that modulate melanin production in response to external factors will likely lead to the development of improved protocols for extraction as well as enhanced yields.

Extracted melanin molecules possess a range of potentially useful applications (**Table 3**). For example, their ability to absorb and dissipate photons from ionizing radiation highlights the potential value of melanin as a sunscreen (Wolbarsht et al., 1981). It has been proposed that due to UV absorption and cytotoxic activities of melanin, they also have therapeutic potential in cancer patients during radiation and chemotherapy treatments (**Table 3**). With an increased consumer demand for natural ingredients in food, melanin has been considered as a natural food coloring. Because the coloration of melanin can range from brown/black (i.e., eumelanin, allomelanin) to red/yellow (i.e., pheomelanin), these melanin molecules could be supplemented in food products as natural color agents instead of synthetic colors (**Table 3**) (Poorniammal et al., 2021; Yang et al., 2023). Various studies have also looked at the potential of melanin as an industrial coating for some packaging materials (**Table 3**).

**Table 3:** Potential uses of fungal melanin.

Species	Applied use	Reference
*Agaricus bisporus*	Protection from UV B	Olaizola et al., 2012
*Agaricus bisporus *	Packaging material	Łopusiewicz et al., 2018a
*Agaricus bisporus *	Antioxidant activity	Łopusiewicz et al., 2018b
*Amorphothe caresinae*	Absorption of both UV A And UV B	Oh et al., 2021
*Aspergillus carbonarius*	Potential yellow food coloring	Narendrababu and Shishupala, 2017
*Auricula auricila-judae*	Protection from ionizing radiation	Revskaya et al., 2012
*Auricularia auricular*	Reduced oxidative stress in mouse liver	Hou et al., 2021
*Blakeslea trispora*	Potential source of β-carotene	Nabae et al., 2005
*Cryptococcus antareticus*	Survival and low mutation rate from space radiation	Onofri et al., 2020
*Gliocephalotrichum simplex*	Reduced oxidative stress in mouse liver	Kunwar et al., 2012
*Monascus purpureus* SM001	Red food coloring	Blanc et al., 1995
*Penicillium aculeatum*	Anticancer drug	Krishnamurthy et al., 2020
*Penicillium europium*	Pink food coloring	Khan et al., 2021
*Talaromyces purpureogenus*	Yellow food coloring	Pandit et al., 2020
*Thermomyces* sp	Food coloring and antioxidant	Poorniammal et al., 2021
*Trichoderma viride*	Brown color pigment	Chitale et al., 2012

## 7 Regulation of melanin synthesis

Although the biosynthetic pathways of melanin production are relatively well known in fungi, less is understood about the regulation of these pathways. However, increasing evidence suggests that the PKA and HOG signaling pathways play a key role in the regulation of melanin production. The PKA-mediated cAMP signaling pathway is highly conserved among fungi, and the regulation of factors involved in virulence via cAMP is quite common (Langfelder et al., 2003; Alspaugh, 2015; Esher et al., 2018). A study by Alspaugh et al. (1997) identified the role of the cAMP-dependent signaling pathway on melanin regulation in *C. neoformans*. A mutant strain with a defect in the Gpa1 Gα-protein, which showed reduced virulence and an inability to synthesize melanin, could be partially complemented by the addition of extracellular 3,5-cyclic adenosine monophosphate (cAMP) (Alspaugh et al., 1997, 1998). This was followed by a study by D’Souza et al. (2001), where the deletion of *pkr1* and *pka1* (two genes encoding the regulatory and catalytic subunits of PKA, respectively) produced avirulent *C. neoformans* strains that lacked melanin production. Other studies highlight a relationship between cAMP signaling and melanin production in plant pathogenic fungi, such as in *Ustilago hordei*, where high levels of cAMP inhibited melanin formation (Lichter and Mills, 1998). Various studies have documented the relationship between the cAMP/PKA pathway and virulence in *C. neoformans* (reviewed by Caza and Kronstad, 2019). In both *Magnaporthe oryzae* and *C. lagenarium*, cAMP signaling is involved in appressoria formation, which uses melanin to facilitate mechanical penetration of the host cell surface during infection (Adachi and Hamer, 1998; Takano et al., 2001). Various studies have also confirmed the importance of the cAMP/PKA signal transduction pathway and its involvement in the expression of genes involved in melanin biosynthesis (Brakhage and Liebmann, 2005; Yu et al., 2017).

A link between glycolysis and melanin production mediated by cAMP/PKA activation in fungi has also been established using mutant strains with defects in genes encoding phosphoglucose isomerase Pgil1 and trehalose synthesis (*TPS1* and *TPS2*) in both *C. neoformans* and *C. gattii* (Ngamskulrungroj et al., 2009; Zhang et al., 2015). These mutants had impaired cAMP/PKA activation with downstream effects on melanin production and the formation of the extracellular capsule (polysaccharide-based capsule). Further evidence of cAMP/pathway involvement was provided by Pukkila-Worley et al. (2005), where *gpa1* mutants had a negative impact on melanin and capsule production in *C. neoformans*, and *cac1* (adenylyl cyclase Cac1) mutants failed to produce melanin and capsules (Choi et al., 2015).

A study looking at transcription factors (TFs) in *C. neoformans* identified four melanin-regulating TFs—*Bzp4*, *Usv101*, *Mbs1*, and *Hob1*—that are required for the induction of the laccase gene (*LAC1*) (Lee et al., 2019). The study found that the cAMP pathway is not involved in the regulation of these four TFs, but the high osmolarity glycerol (HOG) response pathway has a negative impact on the induction of *BZP4* and *LAC1*. The study also focused on various protein kinases and identified Gsk3 and Kic1 deletion mutants as having a negative impact on LAC1 induction (Lee et al., 2019). Overall, this is the most comprehensive study that links specific transcription factors involved in the regulation of melanin synthesis to their cognate upstream signaling pathways.

A recent study determined the effects of protein kinase A (PKA) on *Candida auris* melanization. By performing gene deletion experiments, it was observed that the catalytic subunits Tpk1 and Tpk2 of PKA are important for *C. auris* melanization, whereas Ras1, Gpr1, Gpa2, and Cyr1 are not. Both *tpk1*Δ and *tpk2*Δ mutant strains formed melanin granules, but these melanin granules failed to adhere to the cell wall (Kim and Bahn, 2023). This study showed the importance of PKA catalytic subunits Tpk1 and Tpk2 in the control of chitin synthesis-related genes that are important for melanin granules to adhere to the cell wall. Since the melanin granules are not strongly associated with the cell wall, *C. auris tpk1*Δ *tpk2*Δ mutant strains were more susceptible to oxidative stress compared to the wild-type strain, where both strains produced similar melanin (Kim and Bahn, 2023).

In fungi, mitogen-activated protein kinase (MAPK) signaling pathways play a critical role in many cellular processes, including melanin biosynthesis by perceiving and responding to a variety of stresses or inputs (Gustin et al., 1998). Initial studies identified five MAPK pathways in *Saccharomyces cerevisiae* that were activated by different stimuli (Gustin et al., 1998; Saito, 2010). The orthologs of these five MAPKs have also been determined to play critical roles in different fungi (Turrà et al., 2014). Membrane-spanning proteins such as Sho1, Msb2, Hkr1, Opy2, Sln1, and Ste2 are conserved in fungi and function as sensors that detect stimuli such as osmotic stress, oxidative stress, nutrients, cell wall defects, mating signals, and developmental factors (Turrà et al., 2014; Kou and Naqvi, 2016). In various fungi, the high osmolarity sensitive sensors (i.e., Sho1) activate the HOG-MAPK signaling pathway in response to osmotic stress. In *Verticillium dahliae*, the mutant *ΔSho1* strain showed a reduction in melanin accumulation, and the expression of six genes involved in melanin synthesis was significantly affected (Li et al., 2019).

Evidence of the HOG1 pathway negatively regulating the synthesis of melanin was observed in a study conducted on *C. neoformans* by Bahn et al. (2005), which showed that the *hog1*ΔA mutant strain enhanced capsule formation and had a significant increase in the production of melanin in the serotype A strain H99. To determine the impact of the HOG1 pathway and its relationship with Pka1, it was observed that deletion of the *HOG1* gene resulted in restoring or, in some instances, enhancing the production of melanin in the serotype A *pka1*Δ mutants, suggesting that *HOG1* negatively modulates a downstream target of Pka1 in controlling melanin synthesis (Bahn et al., 2005). A follow-up study by Bahn et al. (2007) demonstrated the effect of a single gene (*SSK2*) encoding an upstream MAPKKK element of the Pbs2-Hog1 MAPK pathway. Ssk2 is known to activate the MAPKK pbs2 via phosphorylation. The *ssk2*Δ mutant strain had an enhanced production of capsules and melanin like the *hog1*Δ mutant, indicating that Ssk2 functions as a key MAPKK controlling the Pbs2-Hog1 MAPK pathway in *C. neoformans* (Bahn et al., 2007).

## 8 Transcription factors regulating fungal melanin production

Recent studies have focused on identifying the TFs involved in the pathways that control melanin biosynthesis. These TFs can either work upstream or downstream of various fungal melanization pathways to influence the expression of genes implicated in melanin production. Shelest (2017) identified 80 TF families in more than 200 fungal species using whole-genome annotation for TFs. Out of the 80 families of TFs, three (i.e., C6Zn clusters, C2H2-like Zn fingers, and homeodomain-like TFs) were generally more prevalent. A previous study conducted by Tsuji et al. (2000) demonstrated the effect of the TFs Cmr1p and Pig1p in *C. lagenarium* and *M. oryzae*, respectively. Both TFs contained C2H2 Zn finger and C6Zn cluster DNA-binding motifs, and deletion of the Zn cluster led to a complete loss of melanin production, whereas deletion of the C2H2 cluster led to reduced melanin production (Tsuji et al., 2000). The TFs involved in the melanization process tend to be conserved across fungi, making them ideal targets to study the process of melanin production.

Cmr1 and its homologs are an example of a TF that is conserved in most melanin-producing fungi. Specifically, it has been shown to regulate genes related to melanin biosynthesis by promoting the expression of the *PKS* gene clusters, thus impacting growth, development, stress response, and virulence in various fungi such as *C. lagenarium*, *M. oryzae*, *Cochliobolus heterostrophus*, *Bipolaris oryzae*, and *Alternaria alternata* (Tsuji et al., 2000; Eliahu et al., 2007; Kihara et al., 2008; Cho et al., 2012; Li et al., 2022). In some fungi, the *PKS* gene and the *CMR1* gene show phylogenetic patterns, suggesting they are subjected to co-evolution (Jia et al., 2021). Evidence of co-evolution (or functional dependency) of the *PKS* and *CMR1* genes has been provided by various knockout studies. In *Alternaria brassicicola*, the *Δamr1* (homolog of cmr1) mutants created melanin-deficient colonies that were more sensitive to UV light (Cho et al., 2012). In *V. dahliae*, both *VdCmr1* and *VdPKS1* were necessary for melanin production, and the *ΔVdCmr1* strain had a 50% reduction in survival when exposed to UV irradiation or high temperatures (40°C) (Wang et al., 2018). In *Botrytis cinerea*, Bcsmr1 was involved in the regulation of genes involved in melanogenesis, and deletion of *bscmr1* led to defects in sclerotial melanogenesis, and an increase in the expression of *bscmr1* led to the accumulation of melanin (Zhou et al., 2017; Schumacher, 2016). In *Setosphaeria turcica*, deletion of the StMR1, a homolog of CMR1, led to the production of lighter colonies, and qPCR analyses confirmed that deletion mutants had significantly decreased expression of six key genes involved in the 1,8-DHN melanin synthesis pathway (Zhang et al., 2022). Another study identified two TFs—Pmr1 (homolog of Cmr1) and Pmr2—that regulate melanin biosynthesis, conidia development, and secondary metabolism in *Pestalotiopsis microspora* (Zhou et al., 2022). The deletion mutant *Δpmr1* showed defects in conidial pigmentation, and the mutant *Δpmr2* had decreased conidial pigmentation (Zhou et al., 2022). In *A. alternata*, the TF Aa*cmrA*, a homolog of cmr1, is required for melanin biosynthesis and pathogenicity. Work on mutant strains *ΔAacmrA* showed severely decreased melanin production, and the mutant strains were more sensitive to oxidative stress and cell wall inhibitors compared to the wild-type strain (Fetzner et al., 2014; Li et al., 2022). These studies provided evidence of the potential co-evolution of the TF Cmr1 and its homologs in various fungi and their involvement in fungal melanization and their effect on PKS.

Besides Cmr1, various other TFs have been identified that play a key role in melanin biosynthesis, some of which are described below. Two genes encoding bHLH (*DevR*) and MADS-box (*Rlm*A) TFs were identified in *A. fumigatus* located upstream of the melanin gene cluster acting as both a repressor and activator of the *pksP* promoter region to modulate the production of conidial melanin (Valiante et al., 2016). Another study identified two TF genes, *PfmaH* and *PfmaF*, that are part of the 1,8-DHN melanin biosynthetic gene cluster (*Pfma*) in *Pestalotiopsis fici* (Zhang et al., 2019). These studies showed that deleting the *PfmaF* did not affect melanin production, but overexpression of *PfmaF* led to heavy pigment accumulation in *P. fici* hyphae (Zhang et al., 2019). In *V. dahliae*, the TF VdMRTF1 is a bZip (basic leucine zipper domain) transcription factor that negatively regulates melanin biosynthesis (Lai et al., 2022). Transcriptomic analysis showed that VdMRTF1 regulates the expression of genes associated with melanin biosynthesis, tyrosine metabolism, and oxidative activity in *V. dahliae* (Lai et al., 2022). Besides BcSMR1, two other TFs—BcZTF1 and BcZTF2—are involved in the regulation of genes involved in melanogenesis in *B. cinerea* (Schumacher, 2016). Overexpression of *bcztf1* and *bcztf2* led to the accumulation of pigmentation in young mycelia, and the deletion mutants *Δbcztf1* and *Δbcztf2* led to colonies appearing white (Schumacher, 2016).

In *C. neoformans*, a GATA-type zinc finger TF (Cir1), which is involved in cAMP/PKA pathway regulation, also showed involvement in melanin and capsule formation (Jung et al., 2006). In another study, Cir1 was shown to regulate two genes involved in the HOG pathway, which is involved in capsule regulation (Haynes et al., 2011). In *C. neoformans*, four TFs (Bzp4, Usv101, Mbs1, and Hob1) were shown to be required for the induction of the laccase gene (*LAC1*) (Lee et al., 2019). Laccases have been shown to play a key role in both 1,8-DHN and L-DOPA melanin (Upadhyay et al., 2013). Another study that demonstrated both the effects of TFs and MAPks was performed on *C. heterostrophus*. In this study, it was found that two mitogen-activated protein kinases (Chk1 and Mps1) were important for normal melanin production (Eliahu et al., 2007). The mutant strains *Δchk* and *Δmps1* both produced white colonies and showed an autolytic appearance. Besides *Δchk* and *Δmps1*, deletion of the CMR1 TFs also resulted in albino mutants and the acquisition of orange-pink coloration, indicating the presence of other carotenoids or secondary metabolites besides melanin in *C. heterostrophus* (Eliahu et al., 2007). **Table 4** summarizes various TFs in fungi and their impact on fungal melanization.

**Table 4:** Transcription factors (TFs) involved in melanin biosynthesis in various melanized fungi.

Transcription factor (TFs)	Impact	Type of melanin	Species	Reference
DevR RlmA	Production of conidial melanin	1,8-DHN	*A. fumigatus*	Valiante et al., 2016
Cmr1/PIG1/BMR1/Amr1 /CmMR1	Regulate melanin biosynthesis	1,8-DHN 1,8-DHN 1,8-DHN 1,8-DHN 1,8-DHN	*C. lagenarium* *M. oryzae* *B. oryzae* *A. brassicicola* *C. minitans*	Eliahu et al., 2007 Tsuji et al., 2000 Kihara et al., 2008 Cho et al., 2012 Luo et al., 2018
PfmaF	Overexpression leads to pigment accumulation	1,8-DHN	*P. fici*	Zhang et al., 2019
Pmr1 and Pmr2	Melanin biosynthesis/ conidia development	1,8-DHN	*P. microspora*	Zhou et al., 2022
AacmrA	Melanin production	1,8-DHN	*A. alternana*	Li et al., 2022
VdCmr1	Melanin biosynthesis/ pigment production	1,8-DHN	*V. dahliae*	Wang et al., 2018
VdMRTF1	Negative melanin regulation	1,8-DHN	*V. dahliae*	Lai et al., 2022
mtf1 and mtf2	Upregulation of PKSs genes	1,8-DHN	*U. maydis*	Reyes-Fernandez et al., 2019
BcSMR1, BcZTF1 and BcZTF2	Regulation of gene involved in melanogenesis	1,8-DHN	*B. cinerea*	Schumacher, 2016
Bzp4, Usv101, Mbs1 and Hob1	Induction of laccase gene (*LAC1*)	L-DOPA	*C. neoformans*	Lee et al., 2019
Cir1	Melanin deposition in the cell wall	L-DOPA	*C. neoformans*	Jung et al., 2006
VdZFP1 and VdZFP2	Involved in melanin deposition	1,8-DHN	*V. dahliae*	Li et al., 2023
Vta1/ VdpF	Involved in melanized microsclerotia development	1,8-DHN	*V. dahliae*	Harting et al., 2020

## 9 Future prospective

Melanin as a biomolecule has been known for over 150 years. Most studies on melanin have focused on its roles in virulence and pathogens that infect humans, animals, and plants. Other research has highlighted the benefits of melanin to fungi, such as protection from environmental stress, which enables them to grow in harsh environments. Melanin pigments are complex polymers whose diversity has made it challenging to investigate their structural properties. However, better extraction protocols coupled with the identification of genes and factors involved in the regulation of melanin biosynthesis have enabled researchers to gain a better understanding of fungal melanization. The biosynthetic pathways for 1,8-DHN melanin and L-DOPA melanin are well known, but recent focus has shifted toward identifying the various TFs and signaling pathways that regulate production. TFs can act as either an activator or a repressor depending on the context in which they bind to their target DNA. Recent work has also provided insight into the regulation of melanin by the cAMP/PKA and the MAPK Hog1 pathways, including links between these pathways and downstream TFs (Cordero et al., 2020; Lee et al., 2019). The level of complexity underlying these links in just one fungus (i.e., *C. neoformans*) suggests that comparable systems-level studies are needed in other fungi such as *E. dermatitidis* to determine the extent, if any, to which regulatory features are conserved.

A more robust mechanistic understanding of the signaling pathways and TFs involved in fungal melanization will help in harnessing the potential benefits of melanin as bio-based components of sunscreens, natural food coloring agents, and packaging materials. These applications have generated more interest in understanding regulatory pathways that can be manipulated to increase the concentration of melanin. In addition, melanin biosynthetic pathways produce many intermediates that have different properties. By genetically modifying strains to hamper or enhance the production of certain intermediates in these biosynthetic pathways, the extraction and yield of the beneficial intermediates can be increased. Another way to increase melanin yield is to genetically modify strains with overexpression of tyrosinases or laccases that play a key role in melanin production. Since melanin production is affected by environmental conditions, experimenting with nutrient composition and growing conditions such as pH, temperature, and aeration can impact the yield of melanin as well.

At this time, it is fair to assume that additional pathways that regulate melanin production beyond those already known remain to be discovered. Systems-level studies that leverage new genetic and genomic-based resources in emerging polyextremotolerant fungi (Erdmann et al., 2022; Carr et al., 2025; Colarusso et al., 2025) represent a promising approach toward addressing this challenge, as do studies that combine classical genetics with genome resequencing (Chhoker et al., 2025). Insights generated by such studies would provide a much more comprehensive understanding of how fungi coordinate melanin production with specific environmental inputs. For example, in those fungi capable of producing multiple types of melanin, do specific inputs direct the synthesis of a particular type of melanin? Obvious benefits derived from these insights include enhanced capacities to engineer melanin production for specific applied purposes. Moreover, they would also create opportunities to delve into broader evolutionary questions regarding the role(s) that the regulation of melanin synthesis might have played in facilitating the adaptation of polyextremotolerant fungi to harsh environmental niches.

## References

[B1] Adachi K. Hamer J. E. (1998). Divergent cAMP signaling pathways regulate growth and pathogenesis in the rice blast fungus *Magnaporthe grisea* . Plant Cell. 10, 1361–1373. doi: 10.2307/3870646, PMID: 9707535 PMC144070

[B2] Alspaugh J. A. (2015). Virulence mechanisms and *Cryptococcus neoformans* pathogenesis. Fungal Genet. Biol. 78, 55–58. doi: 10.1016/j.fgb.2014.09.004, PMID: 25256589 PMC4370805

[B3] Alspaugh J. A. Perfect J. R. Heitmann J. (1997). *Cryptococcus neoformans* mating and virulence are regulated by the G-protein a-subunit GPA1 and cAMP. Genes Dev. 11, 3206–3217. doi: 10.1101/gad.11.23.3206, PMID: 9389652 PMC316752

[B4] Alspaugh J. A. Perfect J. R. Heitmann J. (1998). Signal transduction pathways regulating differentiation and pathogenicity of *Cryptococcus neoformans* . Fungal Genet. Biol. 25, 1–14. doi: 10.1006/fgbi.1998.1079, PMID: 9806801

[B5] Ambrico M. (2016). SPECIAL ISSUE: Melanin, a long lasting history bridging natural pigments and organic bioelectronics. Polym. Int. 65, 1249–1250. doi: 10.1002/pi.5239

[B6] Bahn Y. Geunes-Boyer S. Heitman J. (2007). Ssk2 mitogen-activated protein kinase kinasekinase governs divergent patterns of the stress activated Hog1 signaling pathway in *Cryptococcus neoformans* . Eukaryot. Cell. 6, 2278–2289. doi: 10.1128/ec.00349-07, PMID: 17951522 PMC2168243

[B7] Bahn Y. Kojima K. Cox G. M. Heitman J. (2005). Specialization of the HOG pathway and its impact on differentiation and virulence of *Cryptococcus neoformans* . Mol. Biol. Cell. 16, 2285–2300. doi: 10.1091/mbc.e04-11-0987, PMID: 15728721 PMC1087235

[B8] Baker L. G. Specht C. A. Donlin M. J. Lodge J. K. (2007). Chitosan, the deacetylated form of chitin, is necessary for cell wall integrity in *Cryptococcus neoformans* . Eukaryot. Cell. 6, 855–867. doi: 10.1128/ec.00399-06, PMID: 17400891 PMC1899242

[B9] Banks I. R. Specht C. A. Donlin M. J. Gerik K. J. Levitz S. M. Lodge J. K. (2005). A chitin synthase and its regulator protein are critical for chitosan production and growth of the fungal pathogen *Crypococcus neoformans* . Eukaryot. Cell. 4, 1902–1912. doi: 10.1128/EC.4.11.1902-1912.2005, PMID: 16278457 PMC1287864

[B10] Basarab G. S. Jordan D. B. Gehret T. C. Schwartz R. S. Wawrzak Z. (1999). Design of scytalone dehydratase inhibitors as rice blast fungicides: derivatives of norephedrine. Bioorg. Med. Chem. Lett. 9, 1613–1618. doi: 10.1016/s0960-894x(99)00247-4, PMID: 10386946

[B11] Bayry J. Beaussart A. Dufrêne Y. F. Sharma M. Bansal K. Kniemeyer O.. (2014). Surface structure characterization of *Aspergillus fumigatus* conidia mutated in the melanin synthesis pathway and their human cellular immune response. Infect. Immun. 82, 3141–3153. doi: 10.1128/iai.01726-14, PMID: 24818666 PMC4136205

[B12] Bell A. A. Puhalla J. E. Tolmsoff W. J. Stipanovic R. D. (1976). Use of mutants to establish (+)- scytalone as an intermediate in melanin biosynthesis by *Verticillium dahliae* . Can. J. Microbiol. 22, 787–799. doi: 10.1139/m76-115, PMID: 945120

[B13] Bell A. A. Wheeler M. H. (1986). Biosynthesis and functions of fungal melanins. Ann. Rev. Phytopathol. 24, 411–451. doi: 10.1146/annurev.py.24.090186.002211

[B14] Belozerskaya T. A. Gessler N. N. Aver’yanov A. A. (2016). “Melanin pigments of fungi,” in Fungal Metabolites. Eds. Merillon J.-M. Ramawat K. G. (Springer International Publishing, Cham), 1–29.

[B15] Beltran-Garcia M. J. Prado F. M. Oliveira M. S. Ortiz-Mendoz D. Scalfo A. C. Pesso A.. (2014). Singlet molecular oxygen generation by light activated DHNmelanin of the fungal pathogen *Mycophaerella Fijiensis* in Black Sigatoka Disease of Bananas. PloS One 19, e91616. doi: 10.1371/journal.pone.0091616, PMID: 24646830 PMC3960117

[B16] Bisko N. A. Shcherba V. V. Mitropolskaya N. Y. (2007). Study of melanin complex from medicinal mushroom *Phellinus robustus* . Int. J. Med. Mushrooms. 9, 177–184. doi: 10.1615/IntJMedMushr.v9.i2.80

[B17] Blanc P. J. Loret M. O. Goma G. (1995). Production of citrinin by various species of *Monascus* . Biotechnol. Lett. 17, 291–294. doi: 10.1007/BF01190639

[B18] Bloomfield B. J. Alexander M. (1967). Melanins and resistance of fungi to lysis. J. Bacteriol. 93, 1276–1280. doi: 10.1128/jb.93.4.1276-1280.1967, PMID: 6032507 PMC276597

[B19] Borovanský J. (2011). “History of melanosome research,” in Melanins and Melanosomes (John Wiley & Sons, Hoboken, NJ, USA), 1–19.

[B20] Brakhage A. A. Liebmann B. (2005). *Aspergillus fumigatus* conidial pigment and cAMP signal transduction: significance for virulence. Med. Mycol. 43, S75–S82. doi: 10.1080/13693780400028967, PMID: 16110796

[B21] Butler M. J. Day A. W. (1998). Fungal melanins: a review. Can. J. Microbiol. 44, 1115–1136. doi: 10.1139/w98-119

[B22] Butler M. J. Lazarovits G. Higgins V. J. Lachance M. A. (1989). Identification of a black yeastisolated from oak bark as belonging to genus *Phaecoccomyces* sp. Analysis of melanin produced by the yeast. Can. J. Microbiol. 35, 728–734. doi: 10.1139/m89-118

[B23] Camacho E. Vij R. Chrissian C. Prados-Rosales R. Gil D. O’Meally R. N.. (2019). The structural unit of melanin in the cell wall of the fungal pathogen *Cryptococcus neoformans* . J. Biol. Chem. 294, 10471–10498. doi: 10.1074/jbc.RA119.008684, PMID: 31118223 PMC6615676

[B24] Cao W. Zhou X. McCallum N. C. Hu Z. Ni Z. Kappor U.. (2021). Unraveling the structure and function of melanin through synthesis. J. Am. Chem. Soc 143, 2622–2637. doi: 10.1021/jacs.0c12322, PMID: 33560127

[B25] Carr E. Barton Q. Grambo S. Sullivan M. Renfro C. M. Kuo A.. (2023). Characterization of a novel polyextremotolerant fungus, *Exophiala viscosa*, with insights into its melanin regulation and ecological niche. G3 13, jkad110. doi: 10.1093/g3journal/jkad110, PMID: 37221014 PMC10411609

[B26] Carr E. Bredeweg E. L. Hamilton G. E. Kurbessoian T. Williams A. M. (2025). Pathogenic potential of polyextremotolerant fungi in a warming world. PloS Pathogens. 21, e1013102. doi: 10.1371/journal.ppat.1013102, PMID: 40305547 PMC12043113

[B27] Casadevall A. Nakouzi A. Crippa P. R. Eisner M. (2012). Fungal melanins differ in planar stacking distances. PloS One 7, e30299. doi: 10.1371/journal.pone.0030299, PMID: 22359541 PMC3281024

[B28] Caza M. Kronstad J. W. (2019). The cAMP/Protein Kinase A Pathway regulates virulence and adaptation to host conditions in *Cryptococcus neoformans* . Front. Cell. Infect. Microbiol. 9. doi: 10.3389/fcimb.2019.00212, PMID: 31275865 PMC6592070

[B29] Cecchini M. M. Reale S. Manini P. d’Ischia M. De Angelis F. (2017). Modeling fungal melanin buildup: Biomimetic polymerization of 1,8-Dihydroxynaphthalene mapped by mass spectrometry. Chemistry 23, 8092–8098. doi: 10.1002/chem.201701951, PMID: 28471002

[B30] Chatterjee S. Prados-Rosales R. Itin B. Casadevall A. Stark R. E. (2015). Solid-state NMR reveals the carbon-based molecular architecture of *Cryptococcus neoformans* fungal eumelanins in the cell wall. J. Biol. Chem. 290, 13779–13790. doi: 10.1074/jbc.M114.618389, PMID: 25825492 PMC4447955

[B31] Chen Z. Martinez D. A. Gujja S. Sykes S. M. Zeng Q. Szaniszlo P. J.. (2014). Comparative genomic and transcriptomic analysis of *Wangiells dermatitidis*, a major cause of phaeohyphomycosis and a model black yeast human pathogen. G3 (Bethesda) 4, 561–578. doi: 10.1534/g3.113.009241, PMID: 24496724 PMC4059230

[B32] Chethana K. W. T. Jayawardena R. S. Chen Y. Konta S. Tibpromma S. Abeywickrama P. D.. (2021). Diversity and function of appresoria. Pathogens 10, 746. doi: 10.3390/pathogens10060746, PMID: 34204815 PMC8231555

[B33] Chhoker K. Hausner G. Harris D. (2025). Genetic analysis of pigment production in the fungus *Exophiala dermatitidis* . bioRxiv 2025. doi: 10.1101/2025.03.19.644242 PMC12693494 40902103

[B34] Chitale A. Jadhav D. V. Waghmare S. R. Sahoo A. K. Ranveer R. C. (2012). Production and characterization of brown coloured pigment from *Trichoderma viride* . Electron. J. Environ. Agric. Food Chem. 11, 529–537.

[B35] Cho Y. Srivastava A. Ohm R. A. Lawrence C. B. Wang K. Grigoriev I. V.. (2012). Transcription Factor Amr1 Induces Melanin biosynthesis and suppresses virulence in *Alternaria brassicicola* . PloS Pathog. 8, e1002974. doi: 10.1371/journal.ppat.1002974, PMID: 23133370 PMC3486909

[B36] Choi J. Jung W. H. Kronstad J. W. (2015). The CAMP/Protein kinase A signaling pathway in pathogenic basidiomycete fungi: Connections with iron homeostasis. J. Microbiol. 53, 579–587. doi: 10.1007/s12275-015-5247-5, PMID: 26231374 PMC4697456

[B37] Colarusso A. Williams A. Gladfelter A. S. Wirshing A. C. E. Lew D. J. (2025). Optimized vectors for genetic engineering of *Aureobasidium pullulans* . bioRxiv. 36, mr5. doi: 10.1101/2025.01.25.634885, PMID: 40202829 PMC12206512

[B38] Cordero R. J. B. Camacho E. Casadevall A. (2020). Melanization in *Cryptococcus neoformans* requires complex regulation. mBIO 4, e03313–e03319. doi: 10.1128/mBio.03313-19, PMID: 32019794 PMC7002353

[B39] Cordero R. J. B. Casadevall A. (2017). Functions of fungal melanin beyond virulence. Fungal Biol. Rev. 31, 99–112. doi: 10.1016/j.fbr.2016.12.003, PMID: 31649746 PMC6812541

[B40] Cordero R. J. B. Robert V. Cardinali G. Arinze E. S. Thon S. M. Casadevall A. (2018). Impact of yeast pigmentation on heat capture and latitudinal distribution. Curr. Biol. 28, 2657–2664. doi: 10.1016/j.cub.2018.06.034, PMID: 30078567 PMC6245944

[B41] Costin G. E. Hearing V. J. (2007). Human skin pigmentation: melanocytes modulate skin color in response to stress. FASEB J. 21, 976–994. doi: 10.1096/fj.06-6649rev, PMID: 17242160

[B42] D’Ischia M. Wakamatsu K. Napolitano A. Briganti S. Garcia-Borron J. Kovacs D.. (2013). Melanins and melanogenesis: methods, standards, protocols. Pigment Cell Melanoma Res. 26, 616–633. doi: 10.1111/pcmr.12121, PMID: 23710556

[B43] D’Souza C. A. Alspaugh J. A. Yue C. Harashima T. Cox G. M. Perfect J. R.. (2001). Cyclic AMP-dependent protein kinase controls virulence of the fungal pathogen *Cryptococcus neoformans* . Mol. Cell Biol. 21, 3179–3191. doi: 10.1128/MCB.21.9.3179-3191.2001, PMID: 11287622 PMC86952

[B44] Debler J. W. Henares B. M. (2020). Targeted disruption of scytalone dehydratase gene using *Agrobacterium tumefaciens*-Mediated Transformation leads to altered melanin production in *Ascochyta lentis* . J. Fungi (Basel) 6, 314. doi: 10.3390/jof6040314, PMID: 33255939 PMC7712762

[B45] Ebert M. K. Spanner R. E. Jonge E. Smith D. J. Holthusen J. Secor G. A.. (2018). Gene cluster conservation identifies melanin and perylenequinone biosynthesis pathways in multiple plant pathogenic fungi. Environ. Microbiol. 21, 913–927. doi: 10.1111/1462-2920.14475, PMID: 30421572 PMC7379194

[B46] Eisenman H. C. Casadevall A. (2011). Synthesis and assembly of fungal melanin. Appl. Microbiol. Biotechnol. 93, 931–940. doi: 10.1007/s00253-011-3777-2, PMID: 22173481 PMC4318813

[B47] Eisenman H. C. Casadevall A. (2012). Synthesis and assembly of fungal melanin. Appl. Microbiol. Technol. 93, 931–940. doi: 10.1007/s00253-011-3777-2, PMID: 22173481 PMC4318813

[B48] Eisenman H. C. Frases S. Nicola A. M. Rodrigues M. L. Casadevall A. (2009). Vesicle-associated melanization in *Cryptococcus neoformans* . Microbiology 155, 3860–3867. doi: 10.1128/mbio.03313-19, PMID: 19729402 PMC2889422

[B49] Eisenman H. C. Nosanchuk J. D. Webber J. B. W. Emerson R. J. Camesano T. A. Casadevall A. (2005). Microstructure of cell wall-associated melanin in the human pathogenic fungus *Cryptococcus neoformans* . Biochem 44, 3683–3693. doi: 10.1021/bi047731m, PMID: 15751945

[B50] El-Bialy H. A. El-Gamal M. S. Elsayed M. A. Saudi H. A. Khalifa M. A. (2019). Microbial melanin physiology under stress conditions and gamma radiation protection studies. Radiat. Phys. Chem. 162, 178–186. doi: 10.1016/j.radphyschem.2019.05.002

[B51] Eliahu N. Igbaria A. Rose M. S. Horwitz B. A. Lev S. (2007). Melanin biosynthesis in the maize pathogen *Cochliobolus heterostrophus* depends on two mitogen-activated protein kinases, Chk1 and Mps1, and the transcription factor Cmr1. Eukaryot. Cell. 6, 421–429. doi: 10.1128/ec.00264-06, PMID: 17237364 PMC1828933

[B52] Endo K. Kamo K. Hosono K. Bepu T. Ueda K. (2001). Characterization of mutants defective in melanogenesis and a gene for tyrosinase of *Streptomyces griseus* . J. Antibiot. (Tokyo) 54, 789–796. doi: 10.7164/antibiotics.54.789, PMID: 11776433

[B53] Erdmann E. A. Nitsche S. Gorbushina A. A. Schumacher J. (2022). Genetic engineering of the rock inhabitant *Knufia petricola* provides insight into the biology of extremotolerant black fungi. Front. Fungal. Biol. 3. doi: 10.3389/ffunb.2022.862429, PMID: 37746170 PMC10512386

[B54] Esher S. K. Zaragoza O. Alspaugh J. A. (2018). *Cryptococcal* pathogenic mechanisms: a dangerous trip from the environment to the brain. Mem. Inst. Oswaldo Cruz 113, e180057. doi: 10.1590/0074-02760180057, PMID: 29668825 PMC5909089

[B55] Fetzner R. Seither K. Wenderoth M. Herr A. Fishcer R. (2014). *Alternaria alternata* transcription factor CmrA controls melanization and spore development. Microbiology 160, 1845–1854. doi: 10.1099/mic.0.079046-0, PMID: 24972701

[B56] Fogarty R. V. Tobin J. M. (1996). Fungal melanins and their interactions with metals. Enzyme Microb. Technol. 19, 311–317. doi: 10.1016/0141-0229(96)00002-6, PMID: 8987489

[B57] Franzen A. J. Cunha M. M. Batista E. J. Seabra S. H. de Souza W. Rozental S. (2006). Effects of tricyclazole (5-methyl-1,2,4-triazol[3,4] benzothiazole), a specific DHN-melanin inhibitor, on the morphology of *Fonsecaea pedrosoi* conidia and sclerotic cells. Microsc. Res. Tech. 69, 729–737. doi: 10.1002/jemt.20344, PMID: 16850396

[B58] Fujii I. Mori Y. Watanabe A. Kubo Y. Tsuji G. Ebizuka Y. (2000). Enzymatic synthesis of 1,3,5,8- tetrahydroxynaphthalene solely from malonyl coenzyme Aby a fungal iterative type I Polyketide synthase PKS1. Biochemistry 39, 8853–8858. doi: 10.1021/bi000644j, PMID: 10913297

[B59] Fujii I. Watanabe A. Sankawa U. Ebizuka Y. (2001). Identification of Claisen cyclase domain infungal polyketide synthase WA, a naphthopyrone synthase of *Aspergillus nidulans* . Chem. Biol. 8, 189–197. doi: 10.1016/S1074-5521(00)90068-1, PMID: 11251292

[B60] Funa N. Funabashi M. Ohnishi Y. Horinouchi S. (2005). Biosynthesis of Hexahydroxy perylenequinone melanin via oxidative aryl coupling by cytochrome P-450 in *Streptomyces griseus* . J. Bacteriol. 187, 8149–8155. doi: 10.1128/JB.187.23.8149-8155.2005, PMID: 16291687 PMC1291289

[B61] Gallas J. M. Zajac G. W. Sarna T. Stotter P. L. (2000). Structural differences in unbleached and mildly bleached synthetic tyrosine-derived melanins identified by scanning probe microscopies. Pigment Cell Res. 13, 99–108. doi: 10.1034/j.1600-0749.2000.130208.x, PMID: 10841031

[B62] Gao J. Wenderoth M. Doppler M. Schumacher R. Marko D. Fischer R. (2022). Fungal Melanin biosynthesis pathway as source for fungal toxins. Food Microbiol. 13, e00219–e00222. doi: 10.1128/mbio.00219-22, PMID: 35475649 PMC9239091

[B63] Gessler N. N. Egorova A. S. Belozerskaya T. A. (2014). Melanin pigments of fungi under extreme environmental conditions. Appl. Biochem. Microbiol. 50, 105–113. doi: 10.1134/S0003683814020094 25272728

[B64] Glass K. Ito S. Wilby P. R. Sota T. Nakamura A. Bowers C. R.. (2012). Direct chemical evidence for eumelanin pigment from the Jurassic period. Proc. Natl. Acad. Sci. U.S.A. 109, 10218–10223. doi: 10.1073/pnas.1118448109, PMID: 22615359 PMC3387130

[B65] Gomez B. L. Nosanchuk J. D. (2003). Melanin and fungi. Curr. Opin. Infect. Dis. 16, 91–96. doi: 10.1097/00001432-200304000-00005, PMID: 12734441

[B66] Gorbushina A. A. Kotlova E. R. Sherstneva O. A. (2008). Cellular responses of microcolonial rock fungi to long-term desiccation and subsequent rehydration. Stud. Mycol. 61, 91–97. doi: 10.3114/sim.2008.61.09, PMID: 19287531 PMC2610304

[B67] Gustin M. C. Albertyn J. Alexander M. Davenport K. (1998). MAP kinase pathways in the yeast *Saccharomyces cerevisiae* . Microbiol. Mol. Biol. Rev. 62, 1264–1300. doi: 10.1128/MMBR.62.4.1264-1300.1998, PMID: 9841672 PMC98946

[B68] Hamilton A. J. Gomez B. L. (2002). Melanins in fungal pathogens. J. Med. Microbiol. 51, 189–191. doi: 10.1099/0022-1317-51-3-189, PMID: 11871612

[B69] Harting R. Höfer A. Tran V. T. Weinhold L. M. Barghahn S. Schlüter R.. (2020). The Vta1 transcriptional regulator is required for microsclerotia melanization in *Verticillium dahliae* . Fungal Biol. 124, 490–500. doi: 10.1016/j.funbio.2020.01.007, PMID: 32389312

[B70] Haynes B. C. Skowyra M. L. Spencer S. J. Gish S. R. Williams M. Held E. P.. (2011). Toward an integrated model of capsule regulation in *Cryptococcus neoformans* . PloS Pathogens. 7, e1002411. doi: 10.1371/journal.ppat.1002411, PMID: 22174677 PMC3234223

[B71] Heinekamp T. ThywiBen A. Macheleidt J. Keller S. Valiante V. Brakhage A. A. (2013). *Aspergillus fumigates* melanins: interference with the host endocytosis pathway and impact on virulence. Front. Microbiol. 3, 440. doi: 10.3389/fmicb.2012.00440, PMID: 23346079 PMC3548413

[B72] Henson J. M. Butler M. J. Day A. W. (1999). The dark side of the mycelium: melanins ofphytopathogenic fungi. Annu. Rev. Phytopathol. 37, 447–471. doi: 10.1146/annurev.phyto.37.1.447, PMID: 11701831

[B73] Hong S. Na Y. S. Choi S. Song I. T. Kim W. Y. Lee H. (2012). Non-covalent self-assembly and covalent polymerization co-contribute to polydopamine formation. Adv. Funct. Mater. 22, 4711–4717. doi: 10.1002/adfm.201201156

[B74] Hong S. Wang Y. Park S. Y. Lee H. (2018). Progressive fuzzy cation-π assembly of biological catecholamines. Sci. Adv. 4, eaat7457. doi: 10.1126/sciadv.aat7457, PMID: 30202784 PMC6128673

[B75] Hou R. Liu X. Wu X. Zheng M. Fu J. (2021). Therapeutic effect of natural melanin from edible fungus *Auricularia auricula* on alcohol-induced liver damage *in vitro* and in *vivo* . Food Sci. Hum. Wellness 10, 514–522. doi: 10.1016/j.fshw.2021.04.014

[B76] Jacobson E. S. (2000). Pathogenic roles for fungal melanins. Clin. Microbiol. Rev. 13, 708–717. doi: 10.1128/cmr.13.4.708-717.2000 11023965 PMC88958

[B77] Jia S. Chi Z. Chen L. Liu G. Hu Z. Chi Z. (2021). Molecular evolution and regulation of DHN melanin-related gene clusters are closely related to adaptation of different melanin-producing fungi. Genomics 113, 1962–1975. doi: 10.1016/j.ygeno.2021.04.034, PMID: 33901575

[B78] Jung W. H. Sham A. White R. Kronstad J. W. (2006). Iron regulation of the major virulence factors in the AIDS-associated pathogen *Cryptococcus neoformans* . PloS Biol. 4, e410. doi: 10.1371/journal.pbio.1002410, PMID: 17121456 PMC1637126

[B79] Kejžar A. Gobec S. Plemenitaš A. Lenassi M. (2013). Melanin is crucial for growth of the black yeast *Hortaea werneckii* in its natural hypersaline environment. Fungal Biol. 117, 368–379. doi: 10.1016/j.funbio.2013.03.006, PMID: 23719222

[B80] Keller S. Macheleidt J. Scherlach K. Schmaler-Ripcke J. Jacobsen I. D. Heinekamp T.. (2011). Pyomelanin Formation in *Aspergillus fumigatus* Requires HmgX and the Transcriptional Activator HmgR but Is Dispensable for Virulence. PloS One 10, e26604. doi: 10.1371/journal.pone.0026604, PMID: 22046314 PMC3203155

[B81] Khan A. A. Alshabi A. M. Alqahtani Y. S. Alqahtani A. M. Bennur R. S. Shaikh I. A.. (2021). Extraction and identification of fungal pigment from *Penicillium europium* using different spectral studies. J. King Saud Univ. Sci. 33, 101437. doi: 10.1016/j.jksus.2021.101437

[B82] Kihara J. Moriwaki A. Tanaka N. Tanaka C. Ueno M. Arase S. (2008). Characterization of the BMR1 gene encoding a transcription factor for melanin biosynthesis genes in the phytopathogenic fungus *Bipolaris oryzae* . FEMS Microbiol. Lett. 281, 221–227. doi: 10.1111/j.1574-6968.2008.01101.x, PMID: 18312572

[B83] Kim K. S. Bahn Y. S. (2023). Protein Kinase A controls the melanization of *Candida auris* through the alteration of cell wall components. Antioxid. (Basel) 12, 1702. doi: 10.3390/antiox12091702, PMID: 37760005 PMC10525270

[B84] Kim Y. J. Khetan A. Wu W. Chun S. Viswanathan V. Whitacre J. F.. (2016). Evidence of porphyrin-like structure in natural melanin pigments using electrochemical fingerprinting. Adv. Mater. 28, 3173–3180. doi: 10.1002/adma.201504650, PMID: 26924536

[B85] Koch S. M. Pohl C. F. Siontas O. Cortesao M. Mota A. Runzheimer K.. (2023). *Aspergillus Niger* as a cell factory for the production of pyomelanin, a molecule with UV-C radiation shielding activity. Front. Microbiol. 14. doi: 10.3389/fmicb.2023.1233740, PMID: 37547691 PMC10399693

[B86] Kogej S. Stein M. Volkmann M. Gorbushina A. A. Galinski E. A. Gunde-Cimerman N. (2007). Osmotic adaptation of the halophilic fungus *Hortaeawerneckii*: role of osmolytes and melanization. Microbiology 153, 4261–4273. doi: 10.1099/mic.0.2007/010751-0, PMID: 18048939

[B87] Kou Y. Naqvi N. I. (2016). Surface sensing and signaling networks in plant pathogenic fungi. Sem. Cell Dev. Biol. 57, 84–92. doi: 10.1016/j.semcdb.2016.04.019, PMID: 27133541

[B88] Krishnamurthy S. Narasimha M. K. Thirumale S. (2020). Characterization of ankaflavin from *Penicillium aculeatum* and its cytotoxic properties. Nat. Prod. Res. 34, 1630–1635. doi: 10.1080/14786419.2018.1522633, PMID: 30587035

[B89] Kunwar A. Adhikary B. Jayakumar S. Barik A. Chattopadhyay S. Raghukumar. (2012). Melanin, a promising radioprotector: Mechanisms of actions in a mice model. Toxicol. Appl. Pharmacol. 264, 202–211. doi: 10.1016/j.taap.2012.08.002, PMID: 22968190

[B90] Lai M. Cheng Z. Xiao L. Klosterman S. J. Wang Y. (2022). The bZip transcription factor VdMRTF1 is a negative regulator of melanin biosynthesis and virulence in *Verticillium dahliae* . Microbiol. Spectrum. 10, e0258121. doi: 10.1128/spectrum.02581-21, PMID: 35404080 PMC9045294

[B91] Langfelder K. Stribel M. Jahn B. Haase G. Brakhage A. A. (2003). Biosynthesis of fungal melanins and their importance for human pathogenic fungi. Fungal Genet. Biol. 38, 143–158. doi: 10.1016/s1087-1845(02)00526-1, PMID: 12620252

[B92] Lee D. Jang E. Lee M. Kim S. Lee Y. Lee K.-T.. (2019). Unraveling melanin biosynthesis and signaling networks in *Cryptococcus neoformans* . mBIO 10, e02267. doi: 10.1128/mbio.02267-19, PMID: 31575776 PMC6775464

[B93] Li R. Li Y. Xu W. Zhang M. Jiang Q. Liu Y.. (2022). Transcription factor AacmrA mediated melanin synthesis regulates the growth, appressorium formation, stress response and pathogenicity of pear fungal *Alternaria alternate* . Fungal Biol. 126, 687–695. doi: 10.1016/j.funbio.2022.08.008, PMID: 36116900

[B94] Li H. Sheng R. Zhang C. Wang L. (2023). Two zinc finger proteins, VdZFP1 and VdZFP2, interact with VdCmr1 to promote melanized microsclerotia development and stress tolerance in *Verticillium dahlia* . BMC Biol. 21, 1–22. doi: 10.1186/s12915-023-01697-w, PMID: 37904147 PMC10617112

[B95] Li J. Zhou L. Yin C. Zhang D. Klosterman S. S. J. Wang B.. (2019). The *Verticillium dahliae* Sho1-MAPK pathway regulates melanin biosynthesis and is required for cotton infection. Environ. Microbiol. 21, 4852–4874. doi: 10.1111/1462-2920.14846, PMID: 31667948 PMC6916341

[B96] Lichter A. Mills D. (1998). Control of pigmentation of *Ustilagohordei*: the effect of pH, thiamine, and involvement of the cAMP cascade. Fungal Genet. Biol. 25, 63–74. doi: 10.1006/fgbi.1998.1087, PMID: 9806807

[B97] Lin L. Chen W. (2005). The study of antioxidant effects in melanins extracted from various tissues of animals. Asian-Australas. J. Anim. Sci. 18, 277–281. doi: 10.5713/ajas.2005.277

[B98] Łopusiewicz Ł. Jędra F. Bartkowiak A. (2018a). The application of melanin modified gelatin coatings for packaging and the oxidative stability of pork lard. World Sci. News. 101, 108–119.

[B99] Łopusiewicz Ł. Jędra F. Mizielińska M. (2018b). New Poly (lactic acid) Active packaging composite films incorporated with fungal melanin. Polymers 10, 386. doi: 10.3390/polym10040386, PMID: 30966422 PMC6415272

[B100] Luo C. Zhao H. Yang X. Qiang C. Cheng J. Xie J.. (2018). Functional analysis of the melanin-associated gene *CmMR1* in *Coniothyrium minitans* . Front. Microbiol. 8. doi: 10.3389/fmicb.2018.02658, PMID: 30467498 PMC6237101

[B101] Manini P. Bietti M. Galeotti M. Salamone M. Lanzalunga O. Cecchini M. M.. (2018). Characterization and fate of hydrogen-bonded free-radical intermediates and their coupling products from the hydrogen atom transfer agent 1,8-Naphthalenediol. ACS Omega. 3, 3918–3927. doi: 10.1021/acsomega.8b00155, PMID: 31458630 PMC6641764

[B102] Mattoon E. R. Cordero R. J. B. Casadevall A. (2021). Fungal melanins and applications in healthcare, bioremediation and industry. J. Fungi (Basel) 7, 488. doi: 10.3390/jof7060488, PMID: 34207260 PMC8235761

[B103] Mauch R. M. Cunha V. de. O. Dias A. L. (2013). The copper interference with the melanogenesis of Cryptococcus neoformans. Rev. Inst. Med. Trop. Sao. Paulo. 55, 117–120. doi: 10.1590/s0036-46652013000200009, PMID: 23563765

[B104] Meredith P. Sarna T. (2006). The physical and chemical properties of eumelanin. Pigment Cell Res. 19, 572–594. doi: 10.1111/j.1600-0749.2006.00345.x, PMID: 17083485

[B105] Nabae K. Ishiharam T. Hagiwaram A. Hirota T. Toda Y. Tamano S.. (2005). A 90-day oral toxicity study of beta-carotene derived from *Blakeslea trispora*, a natural food colorant, in F344 rats. Food Chem. Toxicol. 43, 1127–1133. doi: 10.1016/j.fct.2005.03.003, PMID: 15833388

[B106] Nambu N. Tsai H. F. Chang Y. C. Kwon-Chung K. J. (2021). Novel angular naphthopyrone formation by Arp1p dehydratase involved in Aspergillus fumigatus melanin biosynthesis. Environ. Microbiol. Report. 13, 822–829. doi: 10.1111/1758-2229.13013, PMID: 34632721 PMC8612989

[B107] Narendrababu B. Shishupala S. (2017). Spectrophotometric detection of pigments from *Aspergillus* and *Penicillium* isolates. J. App. Biol. Biotechnol. 5, 053–058. doi: 10.7324/JABB.2017.50109

[B108] Natarajan V. T. Ganju P. Ramjumar A. Grover R. Gokhale R. S. (2014). Multifaceted pathways protect human skin from UV radiation. Nat. Chem. Biol. 10, 542–551. doi: 10.1038/nchembio.1548, PMID: 24937072

[B109] Ngamskulrungroj P. Himmelreich U. Breger J. A. Wilson C. Chayakulkeeree M. Krockenberger M. B.. (2009). The trehalose synthesis pathway is an integral part of the virulence composite for *Cryptococcus gattii* . Infect. Immun. 77, 4584–4596. doi: 10.1128/IAI.00565-09, PMID: 19651856 PMC2747965

[B110] Nosanchuk J. D. Casadevall A. (2003). Budding of melanized *Cryptococcus neoformans* in the presence or absence of L-dopa. Microbiology 149, 1945–1951. doi: 10.1099/mic.0.26333-0, PMID: 12855745

[B111] Nosanchuk J. D. Stark R. E. Casadevall A. (2015). Fungal melanin: what do we know about structure? Front. Microbiol. 6. doi: 10.3389/fmicb.2015.01463, PMID: 26733993 PMC4687393

[B112] Oh J. Kim J. Y. Son S. H. Jung W. Kim D. H. Seo J.. (2021). Fungal melanin as a biocompatible broad-spectrum sunscreen with high antioxidant activity. RSC Advances. 11, 19682–19869. doi: 10.1039/d1ra02583j, PMID: 35479243 PMC9033651

[B113] Olaizola C. Abramowski Z. A. Ayala M. J. (2012). Photoprotective effect of fungal melanins against UVB in human skin cells. Mycologia Aplicada Int. 25, 3–12.

[B114] Onofri S. Pacelli C. Selbmann L. Zucconi L. (2020). “The amazing journey of *Cryomyces antarcticus* from Antarctica to space,” in Extremophiles as Astrobiological Models, vol. 2020 . Eds. Seckbach J. Stan-Lotter H. (Wiley, New York, NY, USA), 237–254. doi: 10.1002/9781119593096.ch11

[B115] Ozeki H. Shosuke I. Wakamatsu K. Ishiguro I. (1997a). Chemical characterisation of pheomelanogenesis starting from dihydroxy phenylalanine or tyrosine and cysteine. Effects of tyrosine and cysteine concentrations and reaction time. Biochem. Biophys. Acta 1336, 539–548. doi: 10.1016/s0304-4165(97)00068-8, PMID: 9367182

[B116] Ozeki H. Wakamatsu K. Ito S. Ishiguro I. (1997b). Chemical characterization of eumelanins with special emphasis on 5,6- dihydroxyindole-2-carboxylic acid content and molecular size. Anal. Biochem. 248, 149–157. doi: 10.1006/abio.1997.2079, PMID: 9177734

[B117] Pacelli C. Bryan R. A. Onofri S. Selbmann L. Shuryak I. Dadachova E. (2017). Melanin is effective in protecting fast and slow growing fungi from various types of ionizing radiation. Environ. Microbiol. 4, 1612–1624. doi: 10.1111/1462-2920.13681, PMID: 28127878

[B118] Pandit S. G. Puttananjaiah M. H. Peddha M. S. Dhale M. A. (2020). Safety efficacy and chemical profiling of water-soluble *Talaromyces purpureogenus* CFRM02 pigment. Food Chem. 310, 125869. doi: 10.1016/j.foodchem.2019.125869, PMID: 31771918

[B119] Paolo W. F. Dadachova E. Mandal P. Casadevall A. Szaniszlo P. J. Nosanchuk J. D. (2006). Effects of disrupting the polyketide synthase gene WdPKS1 in *Wangiella [Exophiala] dermatitidis* on melanin production and resistance to killing by antifungal compounds, enzymatic degradation, and extremes in temperature. BMC Microbiol. 6, 55. doi: 10.1186/1471-2180-6-55, PMID: 16784529 PMC1569847

[B120] Perez-Cuesta U. Aparicio-Fernandez L. Guruceaga X. Martin-Souto L. Abad-Diaz-de-Cerio A. Antoran A.. (2020). Melanin and pyomelanin in *Aspergillus fumigatus*: from its genetics to host interaction. Int. Microbiol. 23, 55–63. doi: 10.1007/s10123-019-00078-0, PMID: 31020477

[B121] Pomerantz S. H. Warner M. C. (1967). 3,4-Dihydroxy-L-phenylalanine as the Tyrosinase Cofactor: Occurrence in melanoma and binding constant. J. Biol. Chem. 242, 5308–5314. doi: 10.1016/S0021-9258(18)99429-9, PMID: 4965136

[B122] Poorniammal R. Prabhu S. Dufosse L. Kannan J. (2021). Safety evaluation of fungal pigments for food applications. J. Fungi (Basel) 7, 692. doi: 10.3390/jof7090692, PMID: 34575730 PMC8466146

[B123] Prados-Rosales R. Toriola S. Nakouzi A. Chatterjee S. Stark R. Gerfen G.. (2015). Structural characterization of melanin pigments from commercial preparations of the edible mushroom *Auricularia auricula* . J. Agric. Food Chem. 63, 7326–7332. doi: 10.1021/acs.jafc.5b02713, PMID: 26244793 PMC4862413

[B124] Pukkila-Worley R. Gerrald Q. D. Kraus P. R. Boily M.-J. Davis M. J. Giles S. S.. (2005). Transcriptional network of multiple capsule and melanin genes governed by the *Cryptococcus neoformans* cyclic AMP cascade. Eukaryot. Cell. 4, 190–201. doi: 10.1128/EC.4.1.190-201.2005, PMID: 15643074 PMC544166

[B125] Qin Y. Xia Y. (2024). Melanin in fungi: advances in structure, biosynthesis, regulation, and metabolic engineering. Microb. Cell Factories 23, 334. doi: 10.1186/s12934-024-02614-8, PMID: 39696244 PMC11657710

[B126] Revskaya E. Chu P. Howell R. C. Schweitzer A. D. Bryan R. A. Harris M.. (2012). Compton scattering by internal shields based on melanin-containing mushrooms provides protection of gastrointestinal tract from ionizing radiation. Cancer Biother. Radiopharm. 27, 570–576. doi: 10.1089/cbr.2012.1318, PMID: 23113595 PMC3484786

[B127] Reyes-Fernandez E. Z. Shi Y. Grun P. Bode H. B. Bolker M. (2019). An unconventional melanin biosynthetic pathway in *Ustilago maydis* . Appl. Environ. Microbiol. 87, e01510–e01520. doi: 10.1128/AEM.01510-20, PMID: 33218994 PMC7848912

[B128] Ribera J. Panzarasa G. Stobbe A. Osypova A. Rupper P. Klose D.. (2019). Scalable biosynthesis of melanin by the Basidiomycete *Armillaria cepistipes* . J. Agric. Food Chem. 67, 132–139. doi: 10.1021/acs.jafc.8b05071, PMID: 30541276

[B129] Riesz J. Gilmore J. Meredith P. (2006). Quantitative scattering of melanin solutions. Biophys. J. 90, 4137–4144. doi: 10.1529/biophysj.105.075713, PMID: 16565050 PMC1459487

[B130] Riley P. A. (1997). Melanin. Int. J. Biochem. Cell Biol. 29, 1235–1239. doi: 10.1016/s1357-2725(97)00013-7, PMID: 9451820

[B131] Rosas A. L. Casadevall A. (2001). Melanization decreases the susceptibility of *Cryptococcus neoformans* to enzymatic degradation. Mycopathologia 151, 53–56. doi: 10.1128/IAI.68.6.3696-3703.2000, PMID: 11554578

[B132] Rosas Á. L. Casadevall A. (2006). Melanization affects susceptibility of *Cryptococcus neoformans* to heat and cold1. FEMS Microbiol. Lett. 153, 265–272. doi: 10.1111/j.1574-6968.1997.tb12584.x, PMID: 9271852

[B133] Saito H. (2010). Regulation of cross-talk in yeast MAPK signaling pathways. Curr. Opin. Microbiol. 13, 677–683. doi: 10.1016/j.mib.2010.09.001, PMID: 20880736

[B134] Salgado-Castillo S. N. Lopez-Peria H. A. Diaz R. Pena-Solis K. Ponce-Alquicira E. Soriano-Santos J.. (2023). Fungal melanins and their potential applications: A Review. BioResources 18, 8688–8706. doi: 10.15376/biores.18.4.Castillo

[B135] Sansinenea E. Ortiz A. (2015). Melanin: A solution for photoprotection of *Bacillus thuringiensis* based biopesticides. Biotechnol. Lett. 37, 483. doi: 10.1007/s10529-014-1726-8, PMID: 25381045

[B136] Sapmak A. Boyce K. J. Andrianopoulos A. Vanittanakom N. (2015). The pbrB gene encodes a Laccase required for DHN-melanin synthesis in conidia of *Talaromyces (Penicillium) marneffei* . PloS One. 4, p.e0122728. doi: 10.1371/journal.pone.0122728, PMID: 25866870 PMC4395095

[B137] Schmaler-Ripcke J. Sugareva V. Gebhardt P. Winkler R. Kniemeyer O. Heinekamp T.. (2009). Production of pyomelanin, a second type of melanin, via the tyrosine degradation pathway in *Aspergillus fumigatus* . Appl. Environ. Microbiol. 75, 494–503. doi: 10.1128/AEM.02077-08, PMID: 19028908 PMC2620705

[B138] Schumacher J. (2016). DHN melanin biosynthesis in the plant pathogenic fungus *Botrytis cinerea* is based on two developmentally regulated key enzyme (PKS)-encoding genes. Mol. Microbiol. 99, 729–748. doi: 10.1111/mmi.13262, PMID: 26514268

[B139] Sealy R. C. Hyde J. S. Felix C. C. Menon I. Prota G. (1982). Eumelanins and pheomelanins: characterization by electron spin resonance spectroscopy. Science 217, 545–547. doi: 10.1126/science.6283638, PMID: 6283638

[B140] Seiji M. Fitzpatrick T. B. Simpson R. T. Birbeck M. S. (1963). Chemical composition and terminology of specialized organelles (melanosomes and melanin granules) in mammalian melanocytes. Nature 197, 1082–1084. doi: 10.1038/1971082a0, PMID: 13992623

[B141] Selvakumar P. Rajasekar S. Periasamy K. Raaman N. (2008). Isolation and characterization of melanin pigment from *Pleurotus cystidiosus* . Microbiol. Biotechnol. 24, 2125–2131. doi: 10.1007/s11274-008-9718-2

[B142] Seo D. Choi K.-Y. (2020). Heterologous production of pyomelanin biopolymer using 4-hydroxyphenylpyruvate dioxygenase isolated from *Ralstonia pickettii* in *Escherichia coli* . Biochem. Engr. 157, 107548. doi: 10.1016/j.bej.2020.107548

[B143] Shelest E. (2017). Transcription factors in fungi: TFome dynamics, three major families, and dual-specificity TFs. Front. Genet. 8. doi: 10.3389/fgene.2017.00053, PMID: 28523015 PMC5415576

[B144] Simon J. D. Peles D. N. (2010). The red and the black. Acc. Chem. Res. 43, 1452–1460. doi: 10.1021/ar100079y, PMID: 20734991

[B145] Solano F. (2014). Melanins: skin pigments and much more- types, structural models, biological functions, and formation routes. J. New Sci. 2014, 1–28. doi: 10.1155/2014/498276

[B146] Song W. Yang H. Liu S. Yu H. Li. D. Li P.. (2023). Melanin: insights into structure, analysis, and biological activities for future development. J. Mater. Chem. B. 11, 7528–7543. doi: 10.1039/D3TB01132A, PMID: 37432655

[B147] Stipanovic R. D. Bell A. A. (1976). Pentaketide metabolites of verticillium dahliae 0.3. Identification of (–)-3,4-dihydro-3,8- dihydroxy-1(2h)-naphthalenone [(–)-vermelone] as a precursor to melanin. J. Org. Chem. 41, 2468–2469. doi: 10.1021/jo00876a026, PMID: 945335

[B148] Stüssi H. Rast D. M. (1981). The biosynthesis and possible function of γ-glutaminyl-4-hydroxybenzene in *Agaricus bisporus* . Phytochemistry 20, 2347–2352. doi: 10.1016/S0031-9422(00)82663-1

[B149] Suthar M. Dufosse L. Singh S. K. (2023). The enigmatic world of fungal melanin: A comprehensive Review. J. Fungi (Basel) 9, 891. doi: 10.3390/jof9090891, PMID: 37754999 PMC10532784

[B150] Suwannarach N. Kumla J. Watanabe B. Matsui K. Lumyong S. (2019). Characterization of melanin and optimal conditions for pigment production by an endophytic fungus, *Spissiomyces endophytica* SDBR-CMU319. PloS One 14, e0222187. doi: 10.1371/journal.pone.0222187, PMID: 31498821 PMC6733467

[B151] Takano Y. Komeda K. Kojima K. Okuno T. (2001). Proper regulation of cyclic AMP-dependent protein kinase is required for growth, conidiation, and appressorium function in the anthracnose fungus *Colletotrichum lagenarium* . MPMI 14, 1149–1157. doi: 10.1094/MPMI.2001.14.10.1149, PMID: 11605954

[B152] Thompson J. E. Fahnestock S. Farrall L. Liao D. I. Valent B. Jordan D. B. (2000). The second naphthol reductase of fungal melanin biosynthesis in *Magnaporthe grisea*: tetrahydroxynaphthalene reductase. J. Biol. Chem. 275, 34867–34872. doi: 10.1074/jbc.M006659200, PMID: 10956664

[B153] Tran-Ly A. N. Reyes C. Schwarze F. W. M. R. Ribera J. (2020). Microbial production of melanin and its various applications. World J. Microbiol. Biotechnol. 36, 170. doi: 10.1007/s11274-020-02941-z, PMID: 33043393 PMC7548279

[B154] Tsai H.-F. Wheeler M. H. Chang Y. C. Kwon-Chung K. J. (1999). A developmentally regulated gene cluster involved in conidial pigment biosynthesis in *Aspergillus fumigatus* . J. Bacteriol. 181, 6469–6477. doi: 10.1128/JB.181.20.6469-6477.1999, PMID: 10515939 PMC103784

[B155] Tsirilakis K. Kim C. Vicencio A. G. Christopher A. Casadevall A. Goldman D. L. (2012). Methylxanthine inhibit fungal chitinases and exhibit antifungal activity. Mycopathologia 173, 83–91. doi: 10.1007/s11046-011-9483-x, PMID: 21968902 PMC4289597

[B156] Tsuji G. Kenmochi Y. Takano Y. Sweigard J. Farrall L. Furusawa I.. (2000). Novel fungal transcriptional activators, Cmr1p of *Colletotrichum lagenarium* and pig1p of *Magnaporthe grisea*, contain Cys2His2 zinc finger and Zn(II)2Cys6 binuclear cluster DNA-binding motifs and regulate transcription of melanin biosynthesis genes in a developmentally specific manner. Mol. Microbiol. 38, 940–954. doi: 10.1046/j.1365-2958.2000.02181.x, PMID: 11123670

[B157] Turick C. E. Knox A. S. Becnel J. M. Ekechukwu A. A. Milliken C. E. (2010). Properties and function of pyomelanin. Biopolymers, 449–472. doi: 10.5772/10273

[B158] Turrà D. Segorbe D. Di Pietro A. (2014). Protein kinases in plant-pathogenic fungi: conserved regulators of infection. Annu. Rev. Phytopathol. 52, 267–288. doi: 10.1146/annurev-phyto-102313-050143, PMID: 25090477

[B159] Upadhyay S. Guadalupe T. Xiaorong L. (2013). Laccases involved in 1,8-dihydroxynaphthalene melanin biosynthesis in *Aspergillus fumigatus* are regulated by developmental factors and copper homeostasis. Eukaryot. Cell. 12, 1641–1652. doi: 10.1128/ec.00217-13, PMID: 24123270 PMC3889567

[B160] Upadhyay S. Xu X. Lowry D. Jackson J. C. Roberson R. W. Lin X. (2016). Subcellular compartmentalization and trafficking of the biosynthetic machinery for fungal melanin. Cell Rep. 14, 2511–2518. doi: 10.1016/j.celrep.2016.02.059, PMID: 26972005 PMC4805463

[B161] Valiante V. Baldin C. Hortschansky P. Jain R. (2016). The *Aspergillus fumigatus* conidial melanin production is regulated by the bifunctional bHLHDevR and MADS-box RlmA transcription factors: MADS-box and bHLH transcription factors regulate conidial melanin of *Aspergillus fumigatus* . Mol. Microbiol. 102, 321–335. doi: 10.1111/mmi.13462, PMID: 27393422

[B162] Verde-Yanez L. Usall J. Teixido N. Vall-llaura N. Torres R. (2023). Deciphering the effect of light wavelengths in *Monilinia* spp. DHN-melanin production and their interplay with ROS metabolism in *Monilinia fructicola* . J. Fungi. 9, 653. doi: 10.3390/jof9060653, PMID: 37367589 PMC10304210

[B163] Walker C. A. Gomez B. L. Mora-Montes H. M. Mackenzie K. S. Munro C. A. Brown A. J.. (2010). Melanin externalization in *Candida albicans* depends on cell wall chitin structures. Eukaryot Cell. 9, 1329–1342. doi: 10.1128/EC.00051-10, PMID: 20543065 PMC2937336

[B164] Walton F. J. Idnurm A. Heitman J. (2005). Novel gene functions required for melanization of the human pathogen *Cryptococcus neoformans* . Mol. Microbiol. 57, 1381–1396. doi: 10.1111/j.1365-2958.2005.04779.x, PMID: 16102007

[B165] Wang Y. Hu X. Fang Y. Anchieta A. Goldman P. H. Hernandez G.. (2018). Transcription factor VdCmr1 is required for pigment production, protection from UV irradiation, and regulates expression of melanin biosynthetic genes in *Verticillium dahlia* . Microbiology 164, 685–696. doi: 10.1099/mic.0.000633, PMID: 29485393 PMC5982140

[B166] Wang Z. Zheng L. Hauser M. Becker J. M. Szaniszlo P. J. (1999). WdChs4p, a homolog of chitin synthase 3 in *Saccharomyces cerevisiae*, alone cannot support growth of *Wangiella (Exophiala) dermatitidis* at the temperature of infection. Infect. Immun. 67, 6619–6630. doi: 10.1128/IAI.67.12.6619-6630.1999, PMID: 10569783 PMC97075

[B167] Watanabe A. Fujii I. Tsai H.-F. Chang Y. C. Kwon-Chung K. J. Ebizuka Y. (2000). *Aspergillus fumigatus* alb1 encodes naphthopyrone synthase when expressed in *Aspergillus oryzae* . FEMS Lett. 192, 39–44. doi: 10.1111/j.1574-6968.2000.tb09356.x, PMID: 11040426

[B168] Weijin A. Berg-Somhorst D. B. P. M. Slootweg J. C. Vincken J. P. Gruppen H. Wichers H. J.. (2013). Main phenolic compounds of the melanin biosynthetic pathway in bruising tolerant and bruising sensitive button mushroom (Agaricus bisporus) strains. J. Food Chem. 61, 8224–8231. doi: 10.1021/jf4020558, PMID: 23906106

[B169] Williamson P. R. (1994). Biochemical and molecular characterization of the diphenol oxidase of *Cryptococcus neoformans*: identification as a laccase. J. Bacteriol. 176, 656–664. doi: 10.1128/jb.176.3.656-664.1994, PMID: 8300520 PMC205102

[B170] Wolbarsht M. L. Walsh A. W. George G. (1981). Melanin, a unique biological absorber. Appl. Opt. 20, 2184–2186. doi: 10.1364/AO.20.002184, PMID: 20332914

[B171] Xie W. Pakdel E. Liang Y. Kim Y. J. Liu D. Sun L.. (2019). Natural eumelanin and its derivatives as multifunctional materials for bioinspired applications: A review. Biomacromolecules 20, 4312–4331. doi: 10.1021/acs.biomac.9b01413, PMID: 31696698

[B172] Xu X. Cao R. Li K. Wan Q. Xu G. Lin Y.. (2022). The protective role and mechanism of melanin for *Aspergillus Niger* and *Aspergillus flavus* against chlorine-based disinfectants. Water Res. 223, 119039. doi: 10.1016/j.watres.2022.119039, PMID: 36084430

[B173] Yang F. Cheng L. Du Y. Xia L. Long C. (2022). Functional identification of the DHN melanin synthesis gene cluster and its role in UV-C tolerance in citrus postharvest pathogenic fungus *Penicillium digitatum* . Fungal Biol. 126, 566–575. doi: 10.1016/j.funbio.2022.07.002, PMID: 36008049

[B174] Yang X. Tang C. Zhao Q. Jia Y. Qin Y. Zhang J. (2023). Melanin: A promising source of functional food ingredient. J. Funct. Foods. 105, 105574. doi: 10.1016/j.jff.2023.105574

[B175] Yu X. Liu H. Niu X. Akhberdi O. Wei D. Wang D.. (2017). The Gα1-cAMP signaling pathway controls conidiation, development and secondary metabolism in the taxol-producing fungus *Pestalotiopsis microspora* . Microbiol. Res. 203, 29–39. doi: 10.1016/j.micres.2017.06.003, PMID: 28754205

[B176] Zhang Z. Jia H. Liu N. Li H. Meng Q. Wu N.. (2022). The zinc finger protein StMR1 affects the pathogenicity and melanin synthesis of *Setosphaeria turcica* and directly regulates the expression of DHN melanin synthesis pathway genes. Mol. Microbiol. 117, 261–273. doi: 10.1111/mmi.14786, PMID: 34278632

[B177] Zhang P. Wei D. Li Z. Sun Z. Pan J. Zhu X. (2015). *Cryptococcal* phosphoglucose isomerase is required for virulence factor production, cell wall integrity and stress resistance. FEMS Yeast Res. 15, fov072. doi: 10.1093/femsyr/fov072, PMID: 26271120

[B178] Zhang P. Zhou S. Wang G. An Z. Liu X. Li K.. (2019). Two transcription factors cooperatively regulate DHN melanin biosynthesis and development in *Pestalotiopsis fici* . Mol. Microbiol. 112, 649–666. doi: 10.1111/mmi.14281, PMID: 31116900

[B179] Zhong J. Frases S. Wang H. Casadevall A. Stark R. E. (2008). Following fungal melanin biosynthesis with solid-state NMR: biopolymer molecular structures and possible connections to cell-wall polysaccharides. Biochemistry 47, 4701–4710. doi: 10.1021/bi702093r, PMID: 18370403

[B180] Zhou M. Li Z. Liu Y. Zhang P. Hao X. Zhu X. (2022). Transcription factors Pmr1 and Pmr2 cooperatively regulate melanin biosynthesis, conidia development and secondary metabolism in *Pestalotiopsis microspora* . J. Fungi. 8, 38. doi: 10.3390/jof8010038, PMID: 35049978 PMC8781371

[B181] Zhou Y. Yang L. Wu M. Chen W. Li G. Zhang J. (2017). A Single-Nucleotide Deletion in the Transcription Factor Gene bcsmr1 Causes Sclerotial-Melanogenesis Deficiency in *Botrytis cinerea* . Front. Microbiol. 8. doi: 10.3389/fmicb.2017.02492, PMID: 29312200 PMC5733056

